# Opening up the Valence Shell: A T‐Shaped Iron(I) Metalloradical and Its Potential for Atom Abstraction

**DOI:** 10.1002/anie.202003118

**Published:** 2020-04-27

**Authors:** Jonas C. Ott, Hubert Wadepohl, Lutz H. Gade

**Affiliations:** ^1^ Anorganisch-Chemisches Institut Universität Heidelberg Im Neuenheimer Feld 276 69120 Heidelberg Germany

**Keywords:** iron, ketyl complexes, oxido complexes, paramagnetic NMR spectroscopy, T-shaped complexes

## Abstract

A thermally stable, T‐shaped, d^7^ high‐spin iron(I) complex was obtained by reduction of a PNP‐supported ferrous chloride. Paramagnetic NMR spectroscopy combined with DFT modeling was used to analyze the electronic structure of the coordinatively highly unsaturated complex. The metalloradical character of the compound was demonstrated by the formation of a benzophenone ketyl radical complex upon addition of benzophenone. Furthermore, the compound displays a rich chemistry as an oxygen‐atom abstractor from epoxides, yielding a dinuclear, diferrous [Fe_2_O] complex.

## Graphical abstract

**Radicalized**: A thermally stable, T‐shaped, high‐spin iron(I) PNP complex displayed remarkable reactivity towards oxygen‐containing functional groups, allowing the isolation of a stable iron ketyl radical complex and a bridged, diferrous oxido complex.

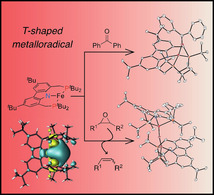

Electronic and coordinative unsaturation leads to enhanced reactivity of transition metal complexes and determines their role as reagents and catalysts.^1^ Iron complexes with coordination numbers of four and larger in both high‐ and low‐spin states are ubiquitous and dominate iron coordination chemistry.^2^ However, with decreasing coordination number and the accompanying electron deficiency at the metal center, the reactivity of these compounds tends to be dramatically increased.^3^ To induce specific reactivity of geometrically well‐defined low‐coordinate complexes, appropriately designed ancillary ligands are required. Especially for the less common iron(I) oxidation state (d^7^), stable compounds have most often required the coordination of additional ligands such as dinitrogen or strong π‐acceptors such as carbon monoxide.^4^ In this context, access to T‐shaped complexes is of special interest, as the vacant coordination site is sterically accessible to small molecules, while other positions at the metal center may be efficiently shielded. However, enforcing a T‐shaped coordination mode can be challenging as three‐coordinate complexes tend to adopt trigonal‐planar (*D*_3*h*_) coordination geometries favored by reduced interligand steric repulsion.^5^

Pincer ligands, with their ligating units of comparable strength, efficiently provide the appropriate arrangement of ligating groups and necessary steric bulk, while leaving unoccupied coordination sites at the metal accessible to substrate binding.^6^ Examples of T‐shaped iron complexes are extremely rare and to the best of our knowledge the only example of such an iron complex bearing a single ancillary ligand was reported by the Caulton group.^7^ We recently demonstrated the ability of the carbazole‐based ligand (PNP)H (with (PNP)H=3,6‐di‐*tert*‐butyl‐1,8‐bis((di‐ *tert*‐butylphosphino)methyl)‐9*H*‐carbazole)^8^, ^9^ to stabilize a series of low‐coordinate 3d metal compounds and induce remarkably slow nuclear relaxation, resulting in unique spectroscopic properties.8a, 10, 11, 12 Herein we report the synthesis of a “naked” (PNP)Fe species, its electronic properties, and its reactivity as a potent oxygen‐atom abstractor.

Nishibayashi and co‐workers recently reported that the reduction of ferrous chlorido complex **1** with KC_8_ under nitrogen atmosphere results in the formation of the dinuclear complex (PNP)Fe‐N≡N‐Fe(PNP).^9^ However, treatment of **1** with excess magnesium powder under argon atmosphere has now led to a dark yellow, paramagnetic product, which was identified as T‐shaped iron(I) complex **2** (Scheme 1). Measurement of the magnetic moment of a C_6_D_6_ solution revealed an effective magnetic susceptibility of 4.2 μ_B_ (Evans method),^13^ indicating a high‐spin *S*=3/2 ground state, which is in accordance with Caulton's previous example.^7^

**Scheme 1:**
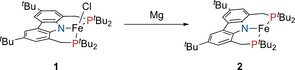
Synthesis of high‐spin (PNP)Fe (**2**) via magnesium reduction of **1**.

The details of the molecular structure of **2** (Figure 1) were established by X‐ray diffraction, which confirmed the open T‐shaped coordination geometry. Its structure was found to be slightly distorted from the idealized coordination geometry, as reflected by the N‐Fe‐P angles of 96°. Notably, the Fe−N bond length of 2.0369(16) Å is longer than that of previously reported Fe^II^ complexes of this ligand, as would be expected for a lower oxidation state of the central metal atom.^10^ Interestingly, no solvent molecule occupies the vacant coordination site of the compound, which was generally found to be relatively inert towards the coordination of pure donor ligands such as ethers or amines.

**Figure 1:**
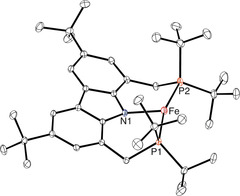
Molecular structure of **2** (displacement ellipsoids drawn at 30 % probability).^25^ Hydrogen atoms are omitted for clarity. Selected bond lengths [Å] and angles [deg]: Fe‐P1 2.2680(6), Fe‐P2 2.2853(6), Fe‐N1 2.0369(16), P1‐Fe‐P2 166.68(2), N1‐Fe‐P1 96.22(5), N1‐Fe‐P2 96.26(5).

To obtain insight into the electronic structure of this remarkable complex, a density functional theory (DFT) study was carried out, using the B3LYP^14^ hybrid density functional with the def2‐TZVP^15^ basis set for the iron atom and the 6‐311G(d,p)^16^ basis set for all other atoms. A plot of the spin density of complex **2** revealed the localization of the majority of unpaired spin around the vacant coordination site, consistent with metalloradical character of the metal center and the assignment of the oxidation state as Fe^I^ already reflected in the metrics of the molecular structure. Furthermore, a strongly negative electrostatic potential at the iron center appears to be in accordance with the chemical inertness of **2** towards σ‐donors such as THF and NEt_3_, with which we were unable to detect any adduct formation.

A closer look into the Kohn–Sham frontier molecular orbitals (MOs) revealed that the LUMO is effectively shielded by the bulky *tert*‐butyl groups, which additionally explains the observed reluctance towards (nonreactive) adduct formation at the vacant coordination site (Figure 2).

**Figure 2:**
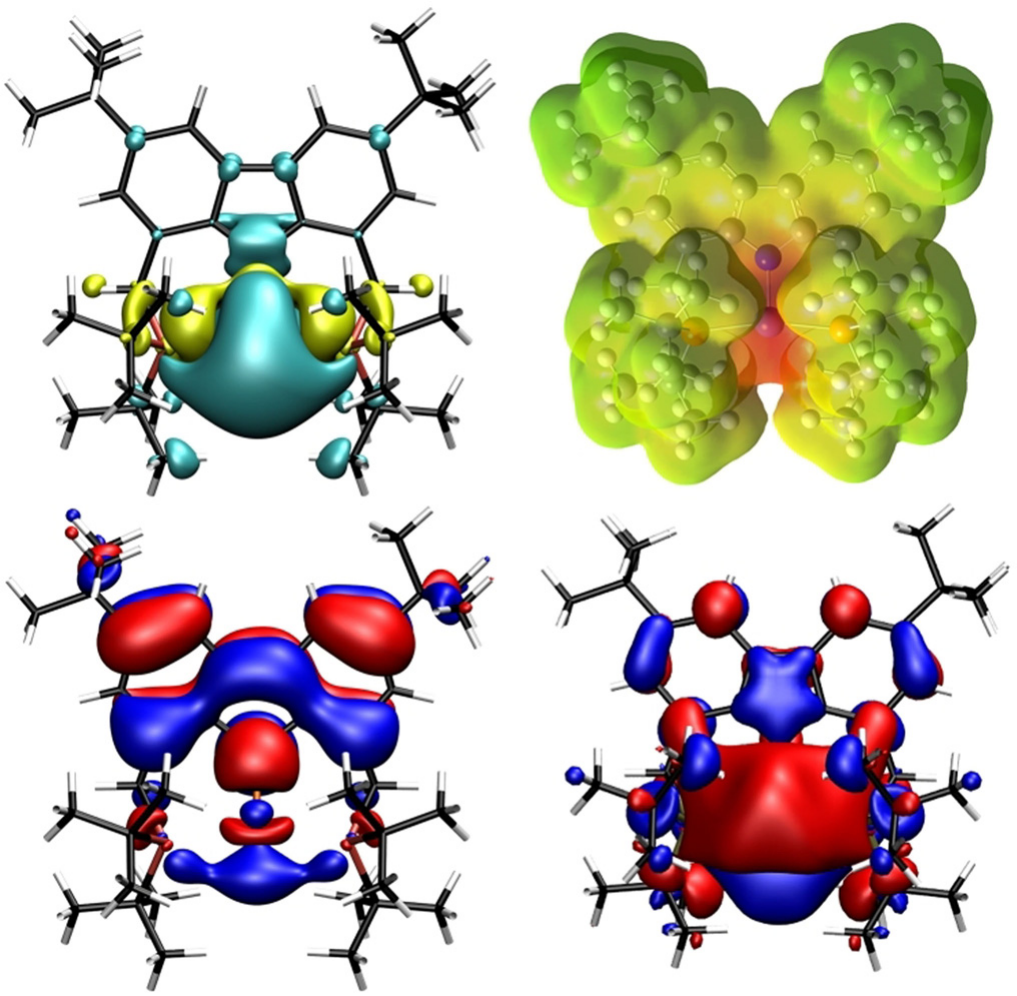
Top left: Distribution of positive (cyan) and negative (yellow) spin density of complex **2** at an isovalue of 0.0004. Top right: Plot of the electrostatic potential of complex **2** ranging from −3.66 (red) to +3.66 (green) at an isovalue of 0.004. Bottom left: MO plot of the highest energy SOMO of complex **2** at an isovalue of 0.02. Bottom right: MO plot of the LUMO of complex **2** at an isovalue of 0.02.

The ^1^H NMR spectrum of **2**, with five signals distributed between +50 and −140 ppm, reflects an effective *C*_2*v*_ symmetry of the molecule in solution (Figure 3). Computational modeling of both contact and pseudocontact contributions to the paramagnetic shifts allowed the full assignment of all proton resonances of **2** as well as the assignment of the corresponding ^13^C NMR resonances (see the Supporting Information). However, in contrast to the complete set of ligand resonances observed for [^*t*Bu^(PNP)FeH],^10^ only those resonances of carbon nuclei were observed in the ^13^C NMR spectrum of the d^7^ high‐spin system **2** with a position more than two bonds away from the paramagnetic center, indicating faster nuclear relaxation rates for the latter compared to the d^6^ intermediate‐spin Fe^II^ hydrido complex.

**Figure 3:**
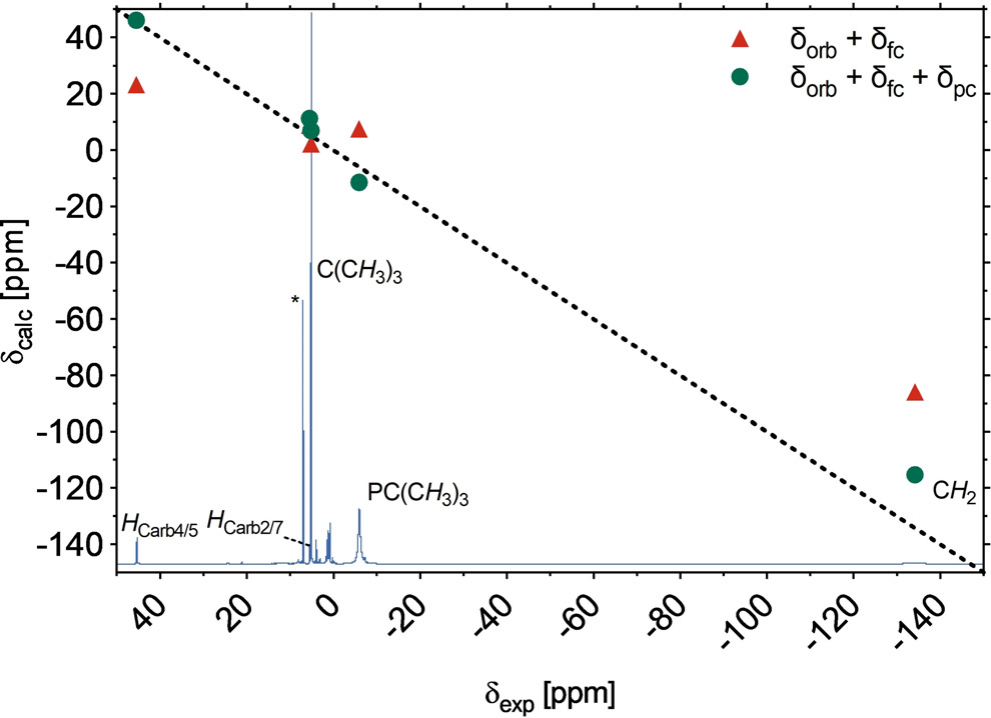
Correlation between the experimental (295 K, 600.13 MHz, C_6_D_6_) and calculated [B3LYP/6‐311G(d,p)+def2‐TZVP(Fe)] proton resonances of complex **2** considering orbital and Fermi‐contact shifts (red triangles) and orbital, Fermi‐contact, and pseudocontact shifts (green circles). The black dotted line represents a perfect correlation of the data (slope *m*=1.0, offset *b*=0.0 ppm). The resonance of the solvent is indicated by an asterisk.

Given the metalloradical character of complex **2** and its resistance to act as a Lewis acid towards σ‐donor ligands, the reactivity towards ligating molecules, which display single electron redox chemistry, was of interest. To probe such behavior, **2** was reacted with benzophenone, giving the thermodynamically stable iron benzophenone ketyl radical complex **3** (Scheme 2). The X‐ray structure analysis of **3** revealed a distorted tetrahedral coordination sphere at the iron center (${\bar{\tau }}_{4}^{{}^{'}}$ =0.70, Figure 4),^17^ which is common for tetracoordinate, high‐spin iron(II) complexes.^10^, ^11^ Whereas Holland et al. recently described an iron η^2^‐benzophenone complex, the end‐on coordination mode of the ketyl ligand in complex **3** has not been reported in iron chemistry.^18^ Analysis of the bond metrics revealed an elongation of the C−O bond length to 1.2989(19) Å [compared to free benzophenone (1.2233(17) Å)],^19^ indicating a decreased bond order, which is in line with a comparable coordinated benzophenone ketyl radical at uranium (1.334(6) Å).^20^ The iron–oxygen bond length of 1.8565(10) Å is within the range of comparable iron alkoxides.^21^

**Figure 4:**
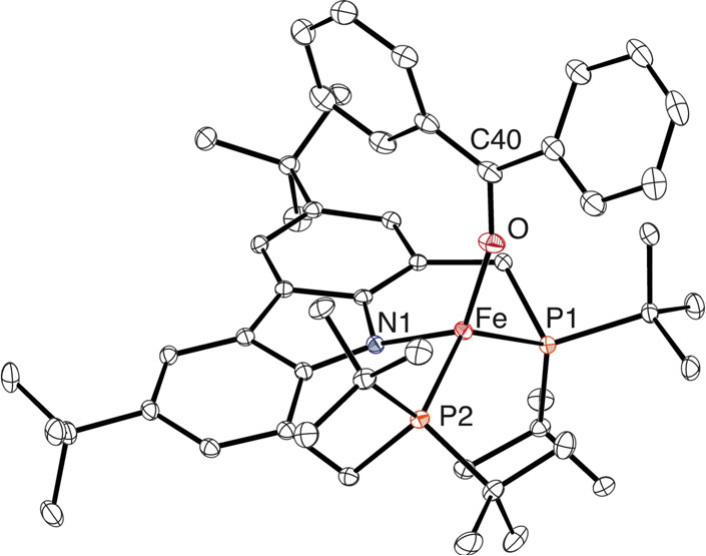
Molecular structure of **3** with displacement ellipsoids drawn at 30 % probability.^25^ Hydrogen atoms are omitted for clarity. Selected bond lengths [Å] and angles [deg]: Fe‐P1 2.4599(4), Fe‐P2 2.3834(4), Fe‐N1 1.9816(11), Fe‐O 1.8565(10), O‐C40 1.2989(18), P1‐Fe‐P2 132.138(14), N1‐Fe‐P1 88.21(3), N1‐Fe‐P2 95.26(3), N1‐Fe‐O 127.75(5), Fe‐O‐C40 158.78(11).

**Scheme 2:**
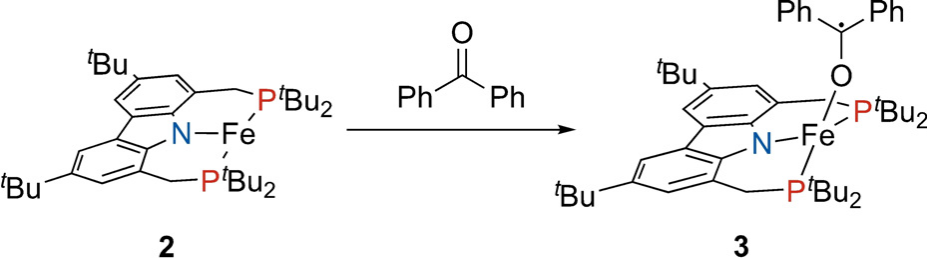
Formation of benzophenone ketyl complex **3** via oxidative addition of benzophenone to **2**.

The magnetic moment of 3.8 μ_B_, as determined for complex **3** by the Evans method,^13^ is consistent with three unpaired electrons, indicating either a high‐spin iron(I) d^7^ system with a coordinated benzophenone or a high‐spin iron(II) d^6^ metal center with an antiferromagnetically coupled ketyl radical. Solid‐state magnetometry furthermore confirmed the quartet ground state of **3** (Figure 5). DFT analysis of complex **3** revealed a high degree of unpaired spin localized on the benzophenone ketyl ligand and, additionally, low‐temperature EPR displayed a distinct singlet resonance with a *g*‐value of 2.0014, indicating the presence of an organic radical. Additionally, we observed an absorption band at 519 nm in the UV/Vis absorption spectrum of complex **3**, which we assign to the π–π* transition of the coordinated ketyl radical ligand fragment. This is within the range of previously reported metal ketyl complexes and explains the purple color of complex **3** (see the Supporting Information).^20^

**Figure 5:**
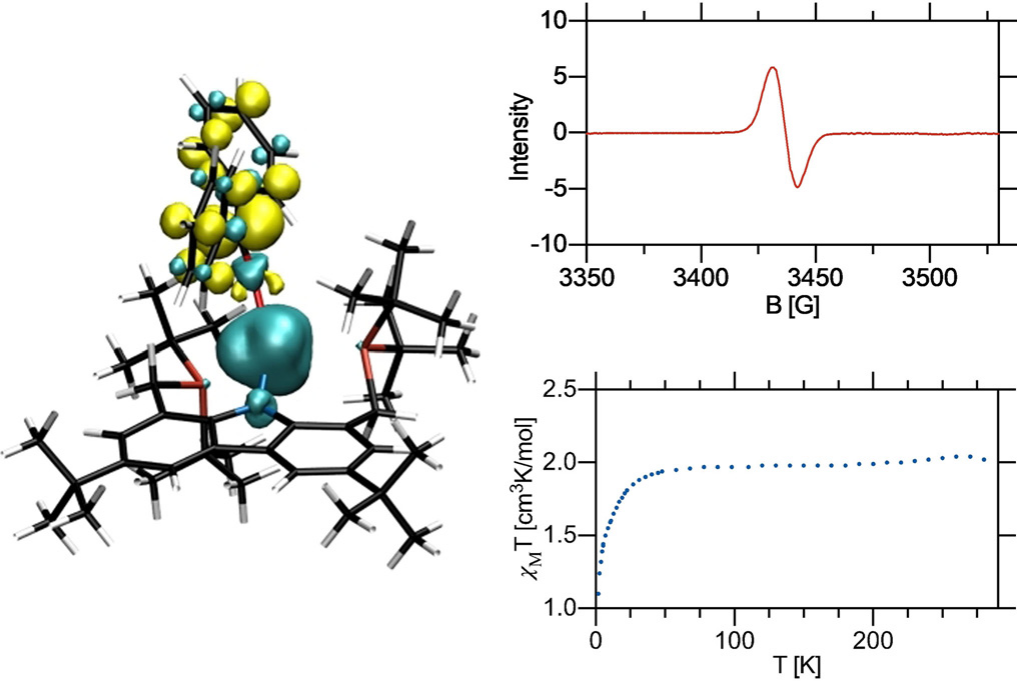
Left: Distribution of positive (cyan) and negative (yellow) spin density of complex **3** at an isovalue of 0.0004. Top right: X‐band electron paramagnetic resonance (EPR) spectrum of complex **3** at 6 K in toluene glass (microwave frequency 9.6374 MHz, *g*=2.0014). Bottom right: Temperature‐dependent SQUID magnetometry of complex **3** recorded at an external field of 1.0 T.

The instantaneous reaction of complex **2** with the reducible benzophenone as an oxygen‐atom donor ligand contrasted with its reluctance to coordinate σ‐donors such as ethers (THF, Et_2_O) or engage in any subsequent transformations. The generation of **3** is thought to be driven by the highly stable alkoxido–Fe^II^ bond, rendering the product thermally stable in solution at 100 °C over periods of days. We therefore hypothesized that using strained cyclic ethers, which may be ring opened, would overcome the apparent inertness of the T‐shaped compound.

Indeed, upon the addition of various epoxides, complex **2** instantaneously and selectively reacted to generate what we identified as a rare example of an oxido‐bridged diferrous complex **4** (Scheme 3).^22^ The molecular structure of **4** was established by X‐ray crystallography and revealed an oxygen‐atom‐bridged structure with both iron centers in a distorted tetrahedral coordination mode (Figure 6), indicated by the structural index parameter ${\bar{\tau }}_{4}^{{}^{'}}$ of 0.74.^17^ The structure comprises a *C*_2_ axis, rendering the two molecular fragments crystallographically equivalent. We note that the paucity of these lower oxidation state oxo Fe^II^Fe^II^ complexes can be seen as a direct consequence of the “oxo wall”.^23^

**Figure 6:**
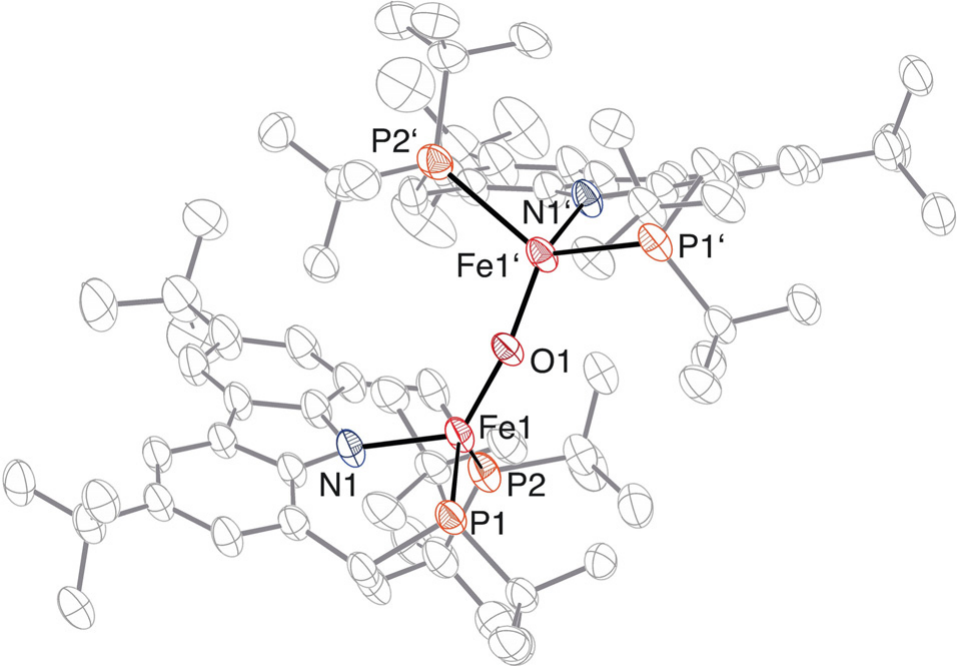
Molecular structure of **4** with displacement ellipsoids drawn at 30 % probability.^25^ Hydrogen atoms are omitted for clarity. Selected bond lengths [Å] and angles [deg]: Fe1‐P1 2.4422(14), Fe1‐P2 2.5323(16), Fe1‐N1 2.012(3), Fe1‐O1 1.7931(9), P1‐Fe1‐P2 127.24(6), N1‐Fe1‐P1 92.91(11), N1‐Fe1‐P2 85.27(12), N1‐Fe1‐O1 127.83(15), Fe1‐N1‐Fe1′ 163.6(2).

**Scheme 3:**
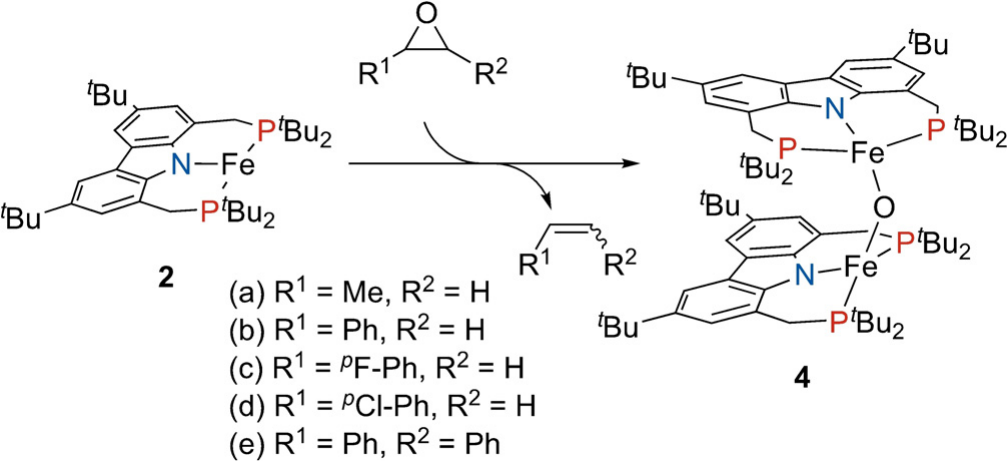
Formation of Fe^II^–Fe^II^ oxo complex **4** via oxygen abstraction from various epoxides.

Interestingly, the formation of complex **4** appears to be unaffected by the nature of the epoxide. We were furthermore able to identify the corresponding alkene as the second reaction product by ^1^H NMR spectroscopy of the reaction mixture. Deoxygenation of *trans*‐ and *cis*‐stilbeneoxide (Scheme 3, e) resulted in a mixture of *trans*‐ and *cis*‐stilbene, which may be due to isomerization via ring‐opened radical species or, subsequently, of the reaction product at the metal center. Additionally, while the use of stronger oxygen‐atom‐transfer reagents such as pyridine *N*‐oxide and trimethylamine *N*‐oxide led to an oxidation of the phosphines, the PNP pincer ligand was unaffected by the presence of an excess of epoxide.

For complex **4** a solution magnetic moment of 3.4 μ_B_ was found (Evans method, [D_8_]toluene, 295 K),^13^ indicating strong antiferromagnetic coupling between the two iron centers. Solid‐state magnetometry (SQUID measurement) revealed an antiferromagnetic coupling constant of *J*_AFC_=−87 cm^−1^, which is in the same range as the *J*_AFC_ value obtained as an estimate from broken symmetry density functional theory (BS‐DFT) of −99.1 cm^−1^.^24^ This antiferromagnetic coupling is also manifested in the variable‐temperature ^1^H NMR experiments in which non‐Curie behavior of the paramagnetic shifts was observed (see the Supporting Information). A complete assignment of the ^1^H NMR resonances for this bridged compound proved to be difficult due to the effective *C*_2_ symmetry in solution, resulting in a complex spectrum with 14 paramagnetically shifted resonances within the limited shift dispersion range of +40 and −25 ppm at 295 K. Nevertheless, an assignment based on relative intensities and ^13^C‐^1^H HETCOR NMR experiments is possible for most resonances.

We have shown that the carbazole‐based PNP pincer ligand developed previously stabilizes a rare example of a highly unsaturated, T‐shaped iron(I) complex, which reacts as a metalloradical species. When it is reacted with benzophenone, an end‐on coordinated iron–benzophenone ketyl radical complex is formed as a consequence of a single electron transfer from the metal to the ligand. To which extent the selective deoxygenation of the “spring‐loaded” epoxides to give the corresponding alkenes also involves radical intermediates remains to be established in future work. Such electron‐transfer‐induced transformations of organic substrates are of considerable synthetic interest.

## Conflict of interest

The authors declare no conflict of interest.

## Supporting information

As a service to our authors and readers, this journal provides supporting information supplied by the authors. Such materials are peer reviewed and may be re‐organized for online delivery, but are not copy‐edited or typeset. Technical support issues arising from supporting information (other than missing files) should be addressed to the authors.

Supplementary
