# Supplementary material for: Opening up the Valence Shell: A T‐Shaped Iron(I) Metalloradical and Its Potential for Atom Abstraction
Source: Angew Chem Int Ed Engl. 2020 Apr 27;59(24):9448–52. doi: 10.1002/anie.202003118 (PMC7318345; doi:10.1002/anie.202003118)
Supplement: Supplementary file 1 — Supplementary [file ANIE-59-9448-s001.pdf]

Angewandte Chemie
Eine Zeitschrift der Gesellschaft Deutscher Chemiker

Supporting Information

Opening up the Valence Shell: A T-Shaped Iron(I) Metalloradical and Its Potential for Atom Abstraction

*Jonas C. Ott, Hubert Wadepohl, and Lutz H. Gade**

anie_202003118_sm_miscellaneous_information.pdf

Supporting Information

S1 Experimental Procedures 2
S2 NMR Spectroscopy 6
S3 Density Functional Theory Calculations..21
S4 SQUID Magnetometry 36
S5 Absorption Spectra..38
S6 EPR Spectra 39
S7 Crystallographic Data 41
S8 Literature 46

S1 Experimental Procedures

S1.1 General Procedures

All experiments were carried out in oven dried glassware under argon atmosphere by using standard glove box or Schlenk techniques. Argon 5.0 from Messer Group GmbH was used as inert gas and dried over Granusic phosphorous pentoxide granulate prior to use. All solvents were purchased anhydrous from Sigma Aldrich and either collected from a solvent purification system (M. Braun SPS 800) after being dried over activated alumina columns or dried and degassed using standard methods and stored in glass ampules under argon atmosphere. Deuterated solvents were purchased from Deutero GmbH, dried over sodium, vacuum distilled, degassed by at least three successive freeze-pump-thaw cycles, and stored over molecular sieves in Teflon valve ampules under argon. Samples for NMR spectroscopy were prepared under argon in 5 mm Wilmad Tubes equipped with J. Young Teflon valves. NMR spectra were recorded on Bruker NMR spectrometers (Avance II 400 MHz, Avance III 600 MHz). ^{1}H and ^{13}C NMR spectra were referenced to residual solvent peaks [C_6D_6 7.16 ppm (^{1}H) and 128.06 ppm (^{13}C)] and are given in parts per million (ppm) relative to external tetramethylsilane. The resonances are reported as chemical shift, multiplicity (*s* singlet, *d* doublet, *t* triplet, *q* quartet, *bs* broad signal), coupling constant *J* and relative integration. UV/Vis spectra were recorded on a Varian Cary 5000 UV-Vis-NIR spectrophotometer at ambient temperature in solutions of *n*-hexane. Elemental analysis (C, H, N) were carried out at the Microanalysis Laboratory of the Faculty of Chemistry and Earth Sciences at the University of Heidelberg with an Elementar Vario MICRO cube machine. EPR spectra were recorded on a Bruker ELEX-SYS-E-500 spectrometer equipped with a ER 041 XK CW microwave bridge. Compound (PNP)FeCl[1] was prepared according to literature. All other reagents were purchased from commercial sources, degassed and used without further purification unless stated otherwise.

S1.2 Preparation of Compounds

S1.2.1 Synthesis of ^{tBu}(PNP)Fe (2)

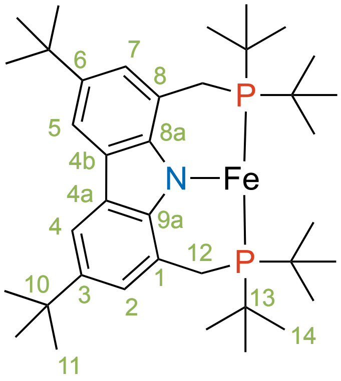

2

A solution of ^{tBu}(PNP)FeCl (**1**) (200.0 mg, 0.29 mmol, 1.0 eq.) in 2 ml THF was added dropwise to a stirring suspension of magnesium powder (35.4 mg, 1.46 mmol, 5.0 eq.) in 1 ml THF at ambient temperature. The suspension was allowed to stir for 12 h, after which the completion of the reaction was indicated by a color change from deep red to dark yellow. The solvent was removed *in vacuo*, the product was taken up in pentane (10 ml) and filtered through a plug of diatomaceous earth. The dark yellow solution was freed of all volatiles *in vacuo* to yield analytically pure **2** as a dark yellow solid (105.4 mg, 0.15 mmol, 53 %.). Single crystals of **2** suitable for X-ray crystallography were obtained as dark yellow crystals by recrystallization of a saturated solution in pentane at –40 °C.

^{1}H NMR (600.13 MHz, C_6D_6, 295 K): δ (ppm) = 45.5 (s, 2H, $H^{Carb4/5}$), 5.7 (s, 2H, $H^{Carb2/7}$), 5.1 (s, 18H, H^{11}, C(CH_3)$_3$), −5.3 (s, 36H, H^{14}, PC(CH_3)$_3$), −132.0 (bs, 4H, H^{12}, CH_2). **^{13}C NMR (150.92 MHz, C_6D_6, 295 K):** δ (ppm) = 582.7 (s, 2C, $C^{Carb4a/4b}$), 372.2 (bs, 2C, C^{14}, PC(CH$_3$)$_3$), 229.4 (s, 2C, $C^{Carb3/6}$), 191.5 (s, 2C, $C^{Carb1/8}$), 110.6 (d, $^1J_{CH}$ = 123.3 Hz, 2C, $C^{Carb4/5}$), 63.1 (q, $^1J_{CH}$ = 125.3 Hz, 2C, C^{11}, C(CH_3)$_3$), 46.2 (d, $^1J_{CH}$ = 154.9 Hz, 2C, $C^{Carb2/7}$), 11.3 (s, 2C, C(CH$_3$)$_3$). **Magnetic Susceptibility:** μ_{eff} = 4.2 μ_B (Evans, C_6D_6, 295 K); μ_{eff} = 4.1 μ_B (SQUID, 290 K). **EA:** Anal. Calcd. for $C_{38}H_{62}FeNP_2$: C, 70.14; H, 9.60; N, 2.15; Found: C, 69.98; H, 10.03; N, 2.27.

S1.2.1 Synthesis of ^{tBu}(PNP)Fe(OCPh$_2$) (3)

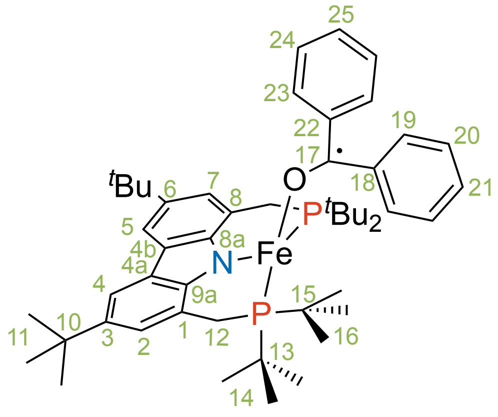

3

A solution of benzophenone (14.0 mg, 0.076 mmol, 1.0 eq.) in toluene was added to a stirring solution of ^{tBu}(PNP)Fe (**2**) (50.0 mg, 0.076 mmol, 1.0 eq.) in 1 ml toluene at ambient temperature and stirred for 1 h. The dark purple solution was freed of all volatiles and the crude product was taken up in pentane and filtered through a plug of diatomaceous earth. The solvent was removed *in vacuo* to yield analytically pure **3** as a dark purple solid (56.0 mg, 0.067 mmol, 88 %). Single crystals of **3** suitable for X-ray crystallography were obtained as dark purple crystals by slow evaporation of a saturated solution of **3** in pentane at ambient temperature.

^{1}H NMR (600.13 MHz, C_6D_6, 295 K): δ (ppm) = 253.0 (s, 2H, $H^{o\text{-Ph}}$), 240.4 (s, 1H, $H^{p\text{-Ph}}$), 223.8 (s, 1H, $H^{p\text{-Ph}}$), 164.6 (s, 2H, C*H*H), 161.2 (s, 2H, $H^{o\text{-Ph}}$), 106.3 (s, 2H, CH*H*), 36.7 (s, 2H, $H^{\text{Carb4/5}}$), 8.2 (s, 18H, PC(CH_3)$_3$), 6.5 (s, 2H, $H^{\text{Carb2/7}}$), 2.1 (s, 18H, PC(CH_3)$_3$), 1.4 (s, 18H, C(CH_3)$_3$), –57.5 (s, 2H, $H^{m\text{-Ph}}$), –70.4 (s, 2H, $H^{m\text{-Ph}}$). **^{13}C NMR (150.92 MHz, C_6D_6, 295 K):** δ (ppm) = 913.8 (s, 2C, $C^{\text{Carb8a/9a}}$), 647.2 (s, 2C, C^{15}, P*C*(CH$_3$)$_3$), 484.6 (s, 2C, $C^{\text{Carb4a/4b}}$), 308.7 (s, 2C, C^{13}, P*C*(CH$_3$)$_3$), 294.9 (s, 2C, $C^{\text{Carb3/6}}$), 251.2 (s, 2C, $C^{\text{Carb1/8}}$), 176.0 (s, 2C, C^{12}, *C*H$_2$), 117.4 (bs, 6C, PC(*C*H$_3$)$_3$), 113.5 (bs, 6C, PC(*C*H$_3$)$_3$), 77.1 (q, $^1J_{CH}$ = 124.7 Hz, 6C, C^{11}, C(*C*H$_3$)$_3$), 56.3 (d, $^1J_{CH}$ = 148.2 Hz, 2C, $C^{\text{Carb4/5}}$), 38.5 (d, $^1J_{CH}$ = 145.7 Hz, 2C, $C^{\text{Carb2/7}}$), –18.5 (s, 2C, C^{10}, *C*(CH$_3$)$_3$). **Magnetic Susceptibility:** μ_{eff} = 3.8 μ_B (Evans, C_6D_6, 295 K). **EA:** Anal. Calcd. for $C_{51}H_{72}FeNOP_2$: C, 73.54; H, 8.71; N, 1.68; Found: C, 73.34; H, 9.00; N, 1.61.

S1.2.2 Synthesis of (^{tBu}(PNP)Fe)$_2$O (4)

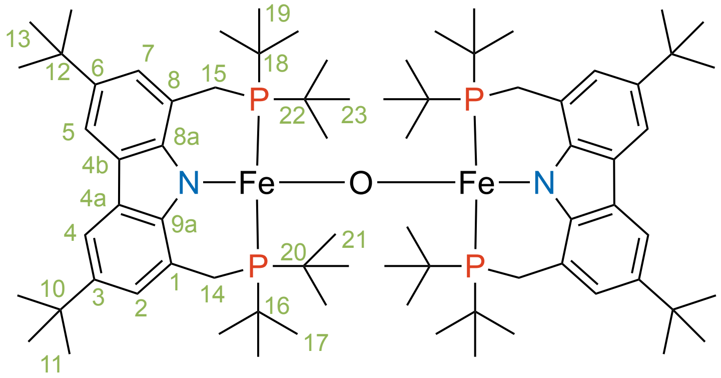

4

To a solution of ^{tBu}(PNP)Fe (**2**) (32.0 mg, 49.18 µmol, 2.0 eq.) in 0.5 ml C_6D_6 was added propylene oxide (2.02 µl, 29.51 µmol, 1.2 eq.) at ambient temperature. Full conversion of the reaction was confirmed by ^{1}H NMR spectroscopy. The solvent was removed *in vacuo*, the crude product was taken up in *n*-hexane and filtered through a plug of diatomaceous earth. The dark yellow solution was freed of all volatiles *in vacuo* to yield **4** as a yellow solid (24.1 mg, 18.29 µmol, 74 %). Single crystals of **4** suitable for X-ray crystallography were obtained as dark yellow crystals by slow evaporation of a saturated solution of **4** in *n*-hexane at ambient temperature.

^{1}H NMR (600.13 MHz, Tol-d$_8$, 295 K): δ (ppm) = 28.5 (s, 2H, CH_2), 22.5 (s, 2H, CH_2), 21.7 (s, 2H, CH_2), 13.1 (s, 2H, $H^{Carb4/5}$), 12.7 (s, 18H, PC(CH_3)$_3$), 12.4 (s, 2H, $H^{Carb4/5}$), 11.4 (s, 18H, PC(CH_3)$_3$), 7.9 (s, 2H, $H^{Carb2/7}$), 2.0 (s, 18H, C(CH_3)$_3$), 0.3 (s, 18H, PC(CH_3)$_3$), −0.4 (s, 18H, C(CH_3)$_3$), −1.1 (s, 2H, $H^{Carb2/7}$), −9.8 (s, 18H, PC(CH_3)$_3$), −56.7 (s, 2H, CH_2). **^{13}C NMR (150.92 MHz, Tol-*d*$_8$, 295 K):** δ (ppm) = 304.8 (s, 2C, C^{Carb}), 259.8 (s, 2C, C^{Carb}), 216.4 (s, 2C, C^{Carb}), 215.0 (s, 2C, C^{Carb}), 168.5 (s, 2C, C^{Carb}), 162.9 (s, 2C, PC(CH$_3$)$_3$), 161.5 (s, 2C, PC(CH$_3$)$_3$), 159.7 (s, 2C, C^{Carb}), 138.7 (s, 2C, C^{Carb}), 135.2 (s, 2C, C^{Carb}), 129.7 (s, 2C, PC(CH$_3$)$_3$), 125.9 (s, 2C, PC(CH$_3$)$_3$), 120.4 (s, 2C, CH$_2$), 113.7 (d, $^1J_{CH}$ = 150.9 Hz, $C^{Carb2/7}$), 104.3 (d, $^1J_{CH}$ = 155.5 Hz, 2C, $C^{Carb4/5}$), 103.2 (d, $^1J_{CH}$ = 155.5 Hz, 2C, $C^{Carb4/5}$), 99.1 (d, $^1J_{CH}$ = 150.9 Hz, 2C, $C^{Carb2/7}$), 56.9 (q, $^1J_{CH}$ = 123.2 Hz, 6C, PC(CH$_3$)$_3$), 54.8 (s, 2C, CH$_2$), 49.4 (q, $^1J_{CH}$ = 123.2 Hz, 6C, PC(CH$_3$)$_3$), 48.7 (q, $^1J_{CH}$ = 125.6 Hz, 6C, PC(CH$_3$)$_3$), 40.8 ((q, $^1J_{CH}$ = 125.0 Hz, 6C, C(CH$_3$)$_3$), 38.3 (q, $^1J_{CH}$ = 125.0 Hz, 6C, C(CH$_3$)$_3$), 33.7 (q, $^1J_{CH}$ = 125.6 Hz, 6C, PC(CH$_3$)$_3$), 28.2 (s, 2C, C(CH$_3$)$_3$), 23.3 (s, 2C, C(CH$_3$)$_3$). **Magnetic Susceptibility:** μ_{eff} = 3.4 μ_B (Evans, C_6D_6, 295 K). **EA:** Anal. Calcd. for $C_{76}H_{124}Fe_2N_2OP_4$: C, 69.29; H, 9.49; N, 2.13; Found: C, 69.55; H, 9.73; N, 2.60.

S2 NMR Spectroscopy

S2.1 Theoretical Background

The chemical shift of a nucleus in paramagnetic compounds may be expressed as the sum of the orbital shift (δ_{orb}) and the hyper fine shift (δ_{hf}) (Eq. S1):[2–4]

$$\delta_{exp} = \delta_{orb} + \delta_{hf} \tag{S1}$$

On the one hand, the orbital shift is temperature independent and is analogous to the diamagnetic contribution to the chemical shift, which is calculated as the difference between the shielding constant of a reference nucleus (in most cases tetramethyl silane is used as a reference substance) and the shielding constant of the nucleus of interest. On the other hand, the hyper fine shift corresponds to the paramagnetic contribution to the chemical shift and is calculated by Eq. S2, where S is the total electron spin of the system, μ_B is the Bohr magneton, k is the Boltzmann constant, T is the temperature, γ_N is the gyromagnetic ratio of the nucleus and the matrices $\boldsymbol{g}$ and $\boldsymbol{A}$ of the g- and hyperfine tensors:[4,5]

$$\delta_{hf} = \frac{S(S+1)\mu_B}{3kT\gamma_N}\boldsymbol{g}.\boldsymbol{A}^{\dagger} \tag{S2}$$

The matrix product of $\boldsymbol{g}$ and the transpose of $\mathbf{A}$ results in a total of four isotropic and five anisotropic terms, which renders the exact determination of the hyperfine shift troublesome. However, in solution NMR the total paramagnetic contribution may be simplified as the sum of the two most significant contributions, which are the Fermi-contact shift (δ_{fc}) and the pseudocontact shift (δ_{pc}), in good approximation (Eq. S3):[5]

$$\delta_{hf} \approx \delta_{fc} + \delta_{pc} \tag{S3}$$

The Fermi-contact shift is temperature dependent and corresponds to the average local magnetic field of the unpaired electrons which in turn is induced by the local density of unpaired spin ρ_N at the nucleus of interest. This spin density is distributed through chemical bonds and may be calculated with the aid of quantum chemistry (Eq. S4), where μ_0 is the vacuum permeability, g_e is the free electron g-factor, k_B is the Boltzmann constant and T is the temperature:[2]

$$\delta_{fc} = \frac{\mu_0\mu_B^2 g_e^2}{9k_B}\frac{(S+1)}{T}\rho_N \tag{S4}$$

On the contrary, the pseudocontact shift represents the dipolar interactions between the magnetic dipoles of the nucleus in question and the unpaired electrons. In d-block metal complexes, the unpaired electrons are, for the most part, located at the metal center, which allows a reasonable determination of the pseudocontact shift contributions by the point-dipole approximation for sufficiently distant nuclei. In molecules, where the magnetic susceptibility is axially anisotropic, the pseudocontact shift can therefore be determined by Eq. S5, with ΔX_{ax} as the axial component of the diagonalized magnetic susceptibility tensor X with $X = X_{mol}/N_A$ [m^3] (where X_{mol} is the molar

susceptibility [m^3mol^{-1}]), r as the length of the vector connecting the nucleus of interest and the unpaired electrons (usually located at the paramagnetic center), and θ is the angle between the r vector and the magnetic field axis.[6]

$$\delta_{pc} = \frac{\Delta\chi_{ax}}{12\pi r^3}(3cos^2\theta - 1) \tag{S5}$$

However, this model fails to predict reliable values for nuclei in close spatial proximity to the unpaired electrons, as the nuclei can no longer be approximated as a set of point dipoles. Furthermore, it follows from Eq. S5 that the extend of the pseudocontact shift highly depends on the spatial distance of the nucleus in question to the unpaired electrons.

S2.2 Assignment of Paramagnetic NMR spectra

Density functional theory (DFT) calculations were employed to evaluate the spin density ρ_n required for the calculation of the Fermi-contact shift (Eq. S4). Additionally, the orbital shift was calculated with the Gauge-Independent Atomic Orbital (GIAO)[7–10] method and is reported with respect to the calculated isotropic shielding constants $\sigma_{TMS}(^1H)$ = 32.03 ppm and $\sigma_{TMS}(^{13}C)$ = 184.53 ppm for tetramethyl silane with the same theoretical conditions after Eq. S6:

$$\delta_{orb} = \sigma_{TMS} - \sigma_{complex} \tag{S6}$$

The pseudocontact shift was initially calculated with the point-dipole approximation (Eq. S5) and the geometry optimized structure of complex **2** in the gas phase was used as a structural model. Furthermore, the magnetic susceptibility tensor was assumed to be axially anisotropic with the magnetic axis incorporating the C_2 symmetry axis along the iron-nitrogen bond. The effective axial magnetic anisotropy was determined to be $\Delta X_{\text{eff, ax,pd}} = 1.35 \times 10^{-31}$ m^3 and succeeds in producing a good correlation between the calculated and the experimental values (Table S1 and S2). To validate the obtained values of this simplified model, the pseudocontact shift was additionally calculated by the integration approach offered by the MATLAB software package SPINACH.[11,12] The resulting pseudocontact shift field (Figure S1) then allowed for the determination of the pseudocontact shift at each nucleus (Table S1 and S2). Interestingly, the obtained effective axial magnetic anisotropy was found to be $\Delta X_{\text{eff, ax,int}} = 1.34 \times 10^{-31}$ m^3, confirming the data obtained by the simplified model. Due to the high effective symmetry of the molecule on the NMR timescale, the approach failed to produce a considerable rhombic component of the magnetic susceptibility.

A theoretical determination of the NMR shifts of compounds **4** was disabled by the complex electronic structure of the compound, and the comparably small chemical shift range of the NMR spectra. However, the assignment of most resonances succeeded based on HETCOR NMR experiments, full width at half maxima (FWHM) and relative intensities of the resonances.

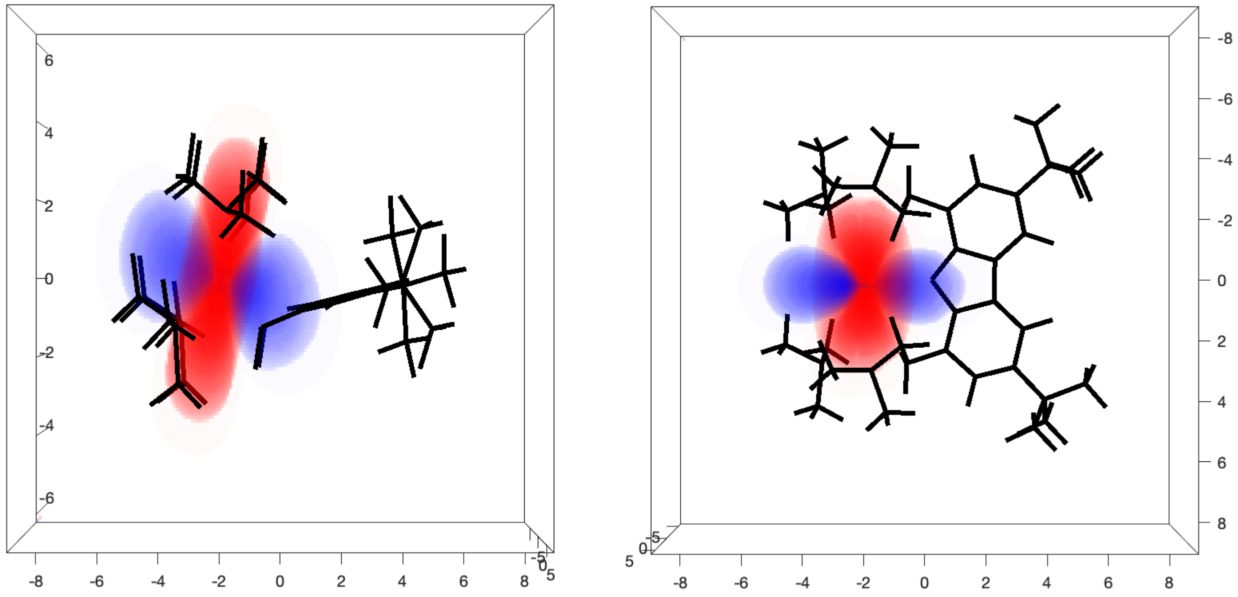

Figure S1. Plot of the pseudocontact shift field obtained from DFT from various perspectives. The dimensions are given in Å. Red: negative pseudocontact shift; Blue: positive pseudocontact shift. Created with the MATLAB software package SPINACH.[12]

The calculated shift δ_{calc} was determined as the sum of the orbital shift (δ_{orb}), the Fermi-contact shift (δ_{fc}) and the pseudocontact shift (δ_{pc}) (Eq. S7).

$$\delta_{calc} = \delta_{orb} + \delta_{fc} + \delta_{pc} \tag{S7}$$

The correlation between the calculated values and the experimentally observed values for the chemical shift of complex **2** and **3** is shown in Figures S2-S4.

Table S1. Assignment of the ^{1}H NMR resonances of complex **2**.

#	δ_{orb} [ppm]	δ_{fc} [ppm]	$\delta_{pc,pd}$ [ppm][a]	$\delta_{pc,int}$ [ppm] [b]	$\delta_{calc,pd}$ [ppm]	$\delta_{calc,int}$ [ppm]	δ_{exp} [ppm] [c]
$H^{2/7}$	6.9	2.0	1.6	2.3	10.5	11.2	5.7
$H^{4/5}$	9.4	13.9	22.5	22.9	45.9	46.2	45.5
H^{11}	1.6	0.6	4.7	4.7	6.9	6.9	5.1
H^{12}	0.3	–86.1	–35.0	–29.4	–120.9	–115.3	–132.0
H^{14}	0.5	7.2	–16.3	–19.1	–8.7	–11.4	–5.3

[a] by point-dipole model; [b] by spin density integration; [c] 295 K, 600.13 MHz, C_6D_6.

Table S2. Assignment of the ^{13}C NMR resonances of complex **2**.

#	δ_{orb} [ppm]	δ_{fc} [ppm]	$\delta_{pc,pd}$ [ppm][a]	$\delta_{pc,int}$ [ppm] [b]	$\delta_{calc,pd}$ [ppm]	$\delta_{calc,int}$ [ppm]	δ_{exp} [ppm] [c]
$C^{1/8}$	125.6	39.8	44.1	36.6	209.5	202.0	191.5
$C^{2/7}$	128.6	−84.6	16.2	19.5	60.1	63.5	46.2
$C^{3/6}$	144.0	55.8	19.3	14.2	219.1	214.0	229.4
$C^{4/5}$	123.1	−24.9	33.5	39.0	131.7	137.2	110.6
$C^{4a/4b}$	136.5	320.6	82.1	76.5	539.3	533.7	582.7
$C^{8a/9a}$	160.8	−182.2	193.5	174.4	172.0	153.0	–[d]
C^{10}	41.9	−23.9	8.2	9.0	26.2	27.0	11.3
C^{11}	34.5	24.6	5.5	5.6	64.6	64.6	63.1
C^{12}	25.5	−1248.7	−45.8	−52.1	−1269.0	−1275.2	–[d]
C^{13}	40.9	−153.3	−54.6	−62.0	−167.0	−174.4	–[d]
C^{14}	30.3	222.6	−26.1	−29.0	226.8	223.8	372.2

[a] by point-dipole model; [b] by spin density integration; [c] 295 K, 150.92 MHz, C_6D_6; [d] not observed.

Table S3. Assignment of the ^{1}H NMR resonances of complex **3**.

#	δ_{orb} [ppm]	δ_{fc} [ppm]	δ_{calc} [ppm]	δ_{exp} [ppm] [a]
$H^{2/7}$	6.9	2.0	12.5	6.5
$H^{4/5}$	8.3	27.9	36.1	36.6
H^{11}	1.5	0.7	2.2	1.4
H^{12}	3.5	90.6	94.2	94.7
$H^{12'}$	3.4	170.3	173.5	173.5
H^{14}	1.1	8.5	9.6	2.1
H^{16}	1.1	10.5	11.6	8.1
H^{19}	9.0	314.7	323.7	161.7
H^{20}	7.6	−135.4	−127.8	−57.8
H^{21}	7.3	356.5	363.8	240.6
H^{23}	5.9	319.6	325.5	253.5
H^{24}	6.3	−132.4	−126.1	−70.5
H^{25}	6.6	338.6	345.2	224.0

[a] 295 K, 600.13 MHz, C_6D_6.

Table S4. Assignment of the ^{13}C NMR resonances of complex **3**.

#	δ_{orb} [ppm]	δ_{fc} [ppm]	δ_{calc} [ppm]	δ_{exp} [ppm] [a]
$C^{1/8}$	126.2	131.4	257.6	251.2
$C^{2/7}$	130.9	-87.6	43.2	38.5
$C^{3/6}$	145.7	125.5	271.2	294.9
$C^{4/5}$	119.9	-50.8	69.1	56.3
$C^{4a/4b}$	132.4	480.0	612.3	484.6
$C^{8a/9a}$	155.1	750.8	905.9	913.8
C^{10}	41.7	−51.8	−10.0	−18.5
C^{11}	34.1	43.5	77.5	77.1
C^{12}	32.1	66.7	98.8	176.0
C^{13}	45.4	299.7	345.1	308.7
C^{14}	31.2	78.3	109.5	113.5
C^{15}	42.9	670.1	713.1	647.2
C^{16}	31.1	93.9	125.1	117.4
C^{17}	182.6	−4220.0	−4037.4	–[b]
C^{18}	147.7	4811.5	4959.2	–[b]
C^{19}	131.9	−1971.6	−1839.7	–[b]
C^{20}	133.9	1615.1	1749.0	–[b]
C^{21}	130.4	−1696.8	−1566.3	–[b]
C^{22}	145.3	4694.0	4839.3	–[b]
C^{23}	128.5	−1978.6	−1850.1	–[b]
C^{24}	133.1	1540.4	1673.5	–[b]
C^{25}	131.3	−1617.1	−1485.8	–[b]

[a] 295 K, 150.92 MHz, C_6D_6; [b] not observed.

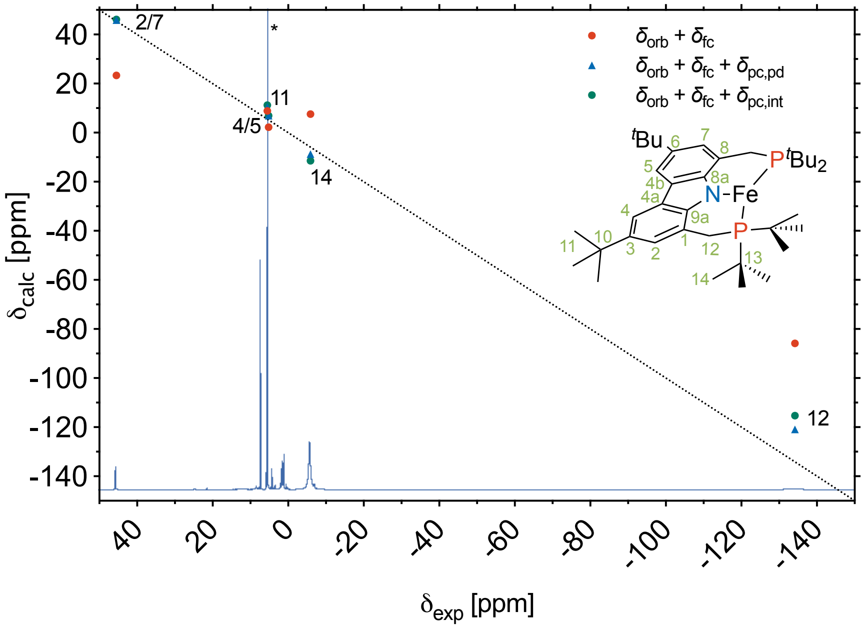

Figure S2. Plot of the correlation between the experimental (295 K, 600.13 MHz, C_6D_6) and calculated [B3LYP/6-311G(d,p) + def2-TZVP (only for iron)] ^{1}H NMR resonances of complex **2**. The dotted line represents a perfect correlation (m = 1.0, y = 0.0). Solvent resonances are indicated with an asterisk.

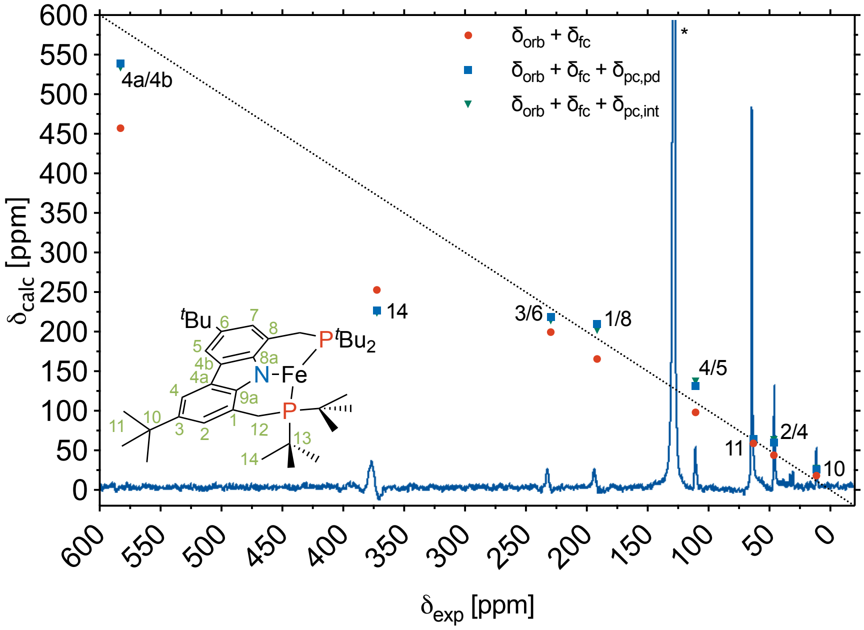

Figure S3. Plot of the correlation between the experimental (295 K, 150.92 MHz, C_6D_6) and calculated [B3LYP/6-311G(d,p) + def2-TZVP (only for iron)] ^{13}C NMR resonances of complex **2**. The dotted line represents a perfect correlation (m = 1.0, y = 0.0). Solvent resonances are indicated with an asterisk.

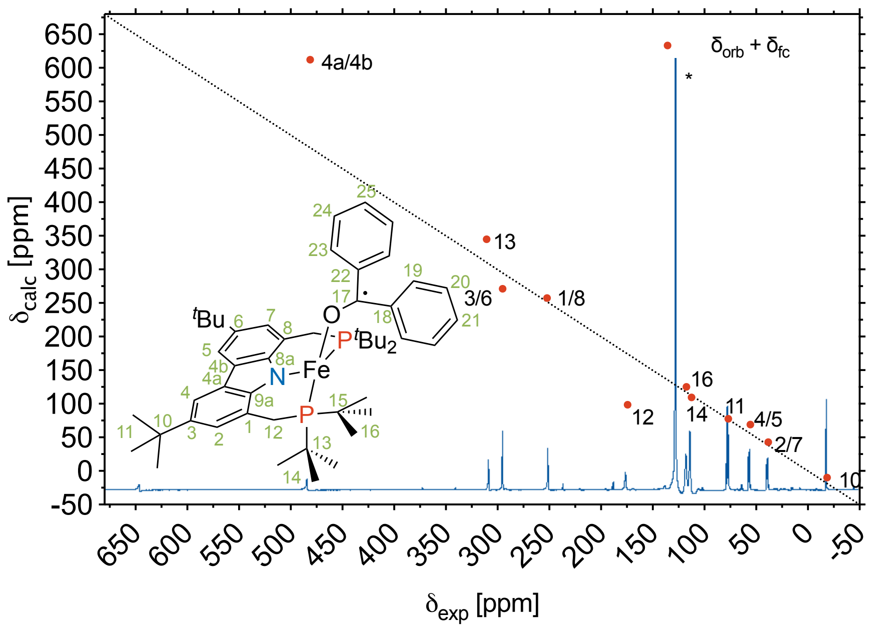

Figure S4. Plot of the correlation between the experimental (295 K, 150.92 MHz, C_6D_6) and calculated [B3LYP/6-311G(d,p) + def2-TZVP (only for iron)] ^{13}C NMR resonances of complex **3**. The dotted line represents a perfect correlation (m = 1.0, y = 0.0). Solvent resonances are indicated with an asterisk.

S2.3 General Acquisition Procedure

Unless stated otherwise, all spectra were recorded at a probe temperature of 295 K. Assignment of the signals was persecuted with the aid of HETCOR NMR experiments. The data acquisition parameters used for the paramagnetic ^{1}H, ^{2}H, ^{13}C and HETCOR NMR experiments are displayed in Table S5. All ^{13}C data were recorded on a Bruker Avance III 600 MHz spectrometer equipped with a He-cooled Cryo Probe. All temperature dependent ^{1}H spectra were recorded on a Bruker Avance II 400 MHz spectrometer (Table S6). All spectra were processed with the Bruker NMR software TopSpin 4.0.6.

Table S5. Acquisition parameters for paramagnetic ^{1}H, ^{13}C and HETCOR NMR experiments at Bruker AVIII 600.

Parameter	^{1}H	^{13}C	HETCOR
Pulse program (Bruker)	zg	zg	hxcoqf
90° pulse width *p*1 [*μ*s]	14.0	12.0	12.0 (^{13}C)
Pulse width used [°] / [*μ*s]	9 / 1.4	60 / 8.0	90 / 12.0 (^{13}C)
Sweep width *F*1 [ppm]	1500	2000	300
Sweep width *F*2 [ppm]			200
Acquisition data points *td*	64k	98k	8k × 128
Resulting acquisition time *aq* [s]	0.055	0.157	0.09 | 0.0005
Relaxation delay *d*1 [s]	0.01	0.05	0.05
Preaquisition delay *de* [*μ*s]	6.5	18.0	18.0
Number of scans *ns*	4k	64k	128

Table S6. Acquisition parameters for paramagnetic NMR experiments at Bruker AVII 400.

Parameter	^{1}H
Pulse program (Bruker)	zg
90° pulse width *p*1 [*μ*s]	14.0
Pulse width used [°] / [*μ*s]	9 / 1.4
Sweep width *F*1 [ppm]	800
Acquisition data points *td*	64k
Resulting acquisition time *aq* [s]	0.1
Relaxation delay *d*1 [s]	0.05
Preaquisition delay *de* [*μ*s]	6.5
Number of scans *ns*	2k

S2.4 NMR Spectra

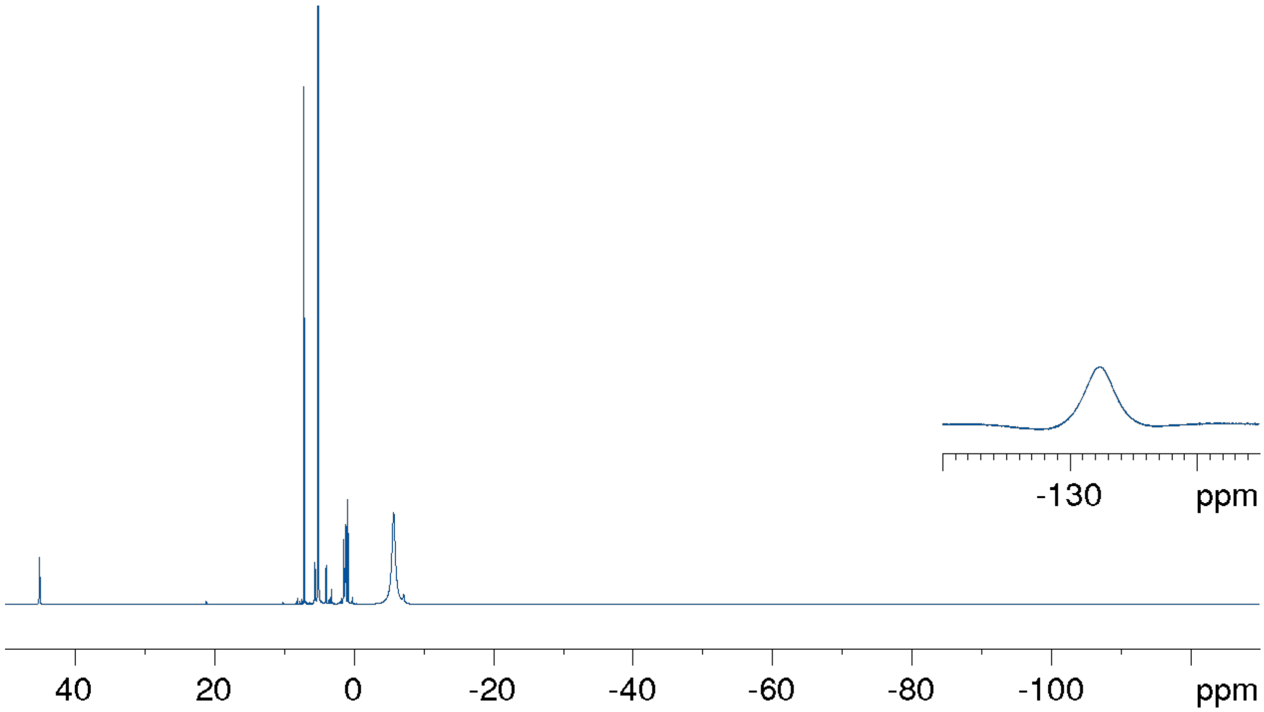

Figure S5. ^{1}H NMR spectrum (600.13 MHz, C_6D_6, 295 K) of complex **2**.

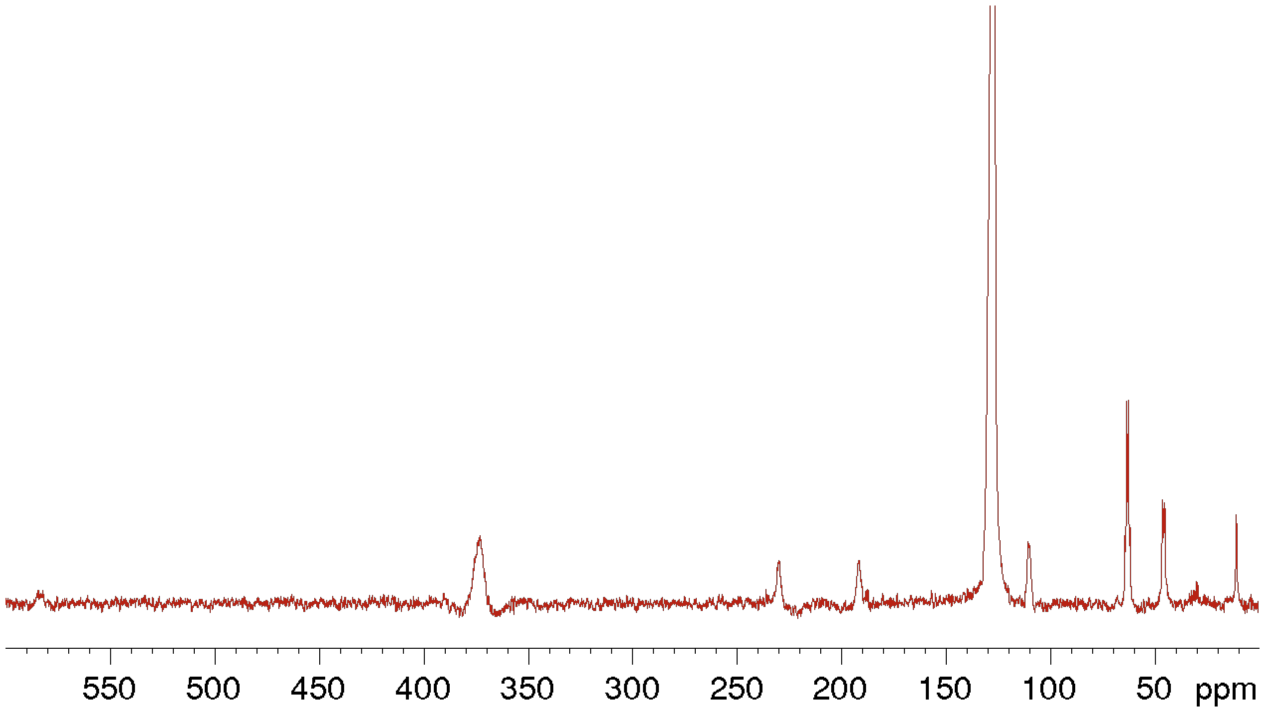

Figure S6. ^{13}C NMR spectrum (150.92 MHz, C_6D_6, 295 K) of complex **2**.

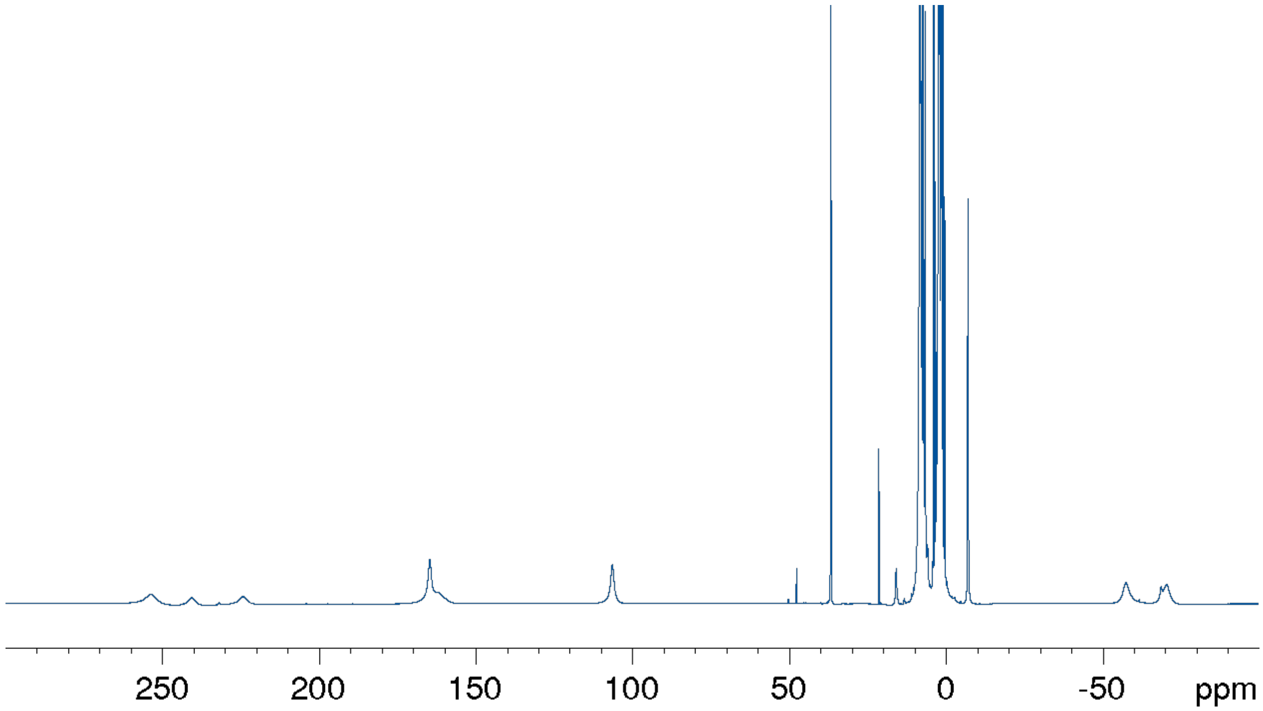

Figure S7. ^{1}H NMR spectrum (600.13 MHz, C_6D_6, 295 K) of complex **3**.

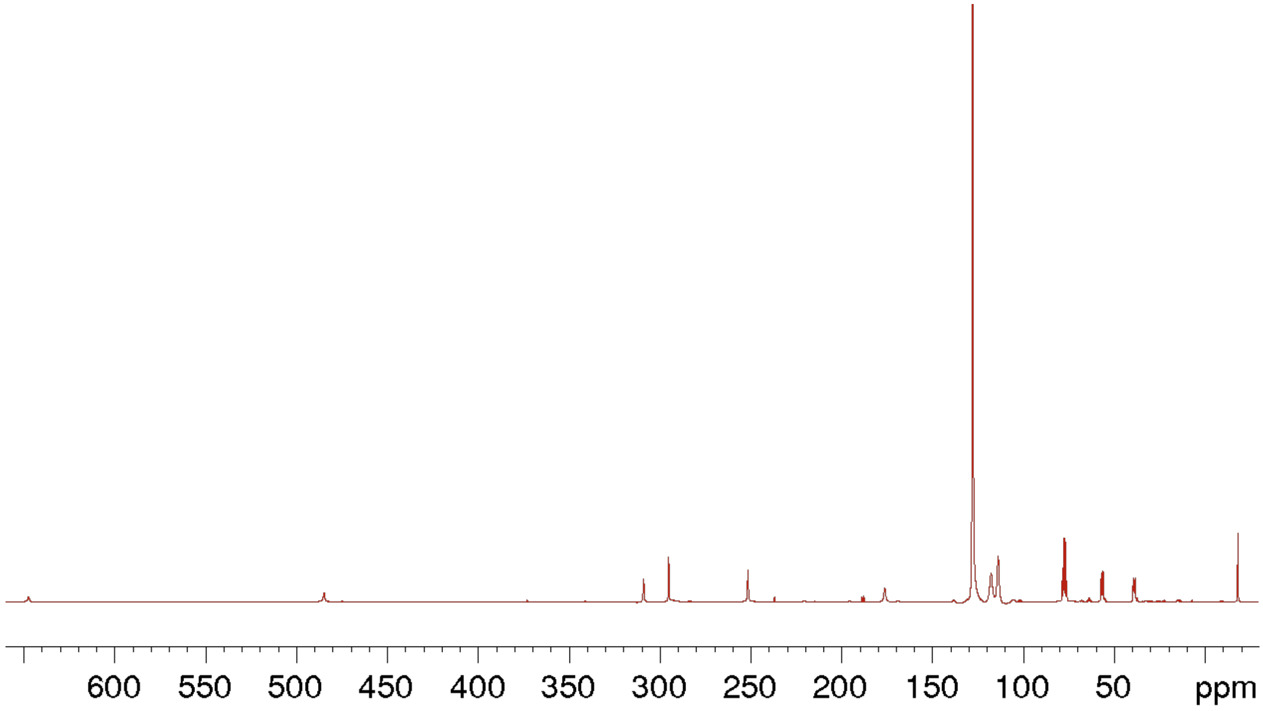

Figure S8. ^{13}C NMR spectrum (150.92 MHz, C_6D_6, 295 K) of complex **3**.

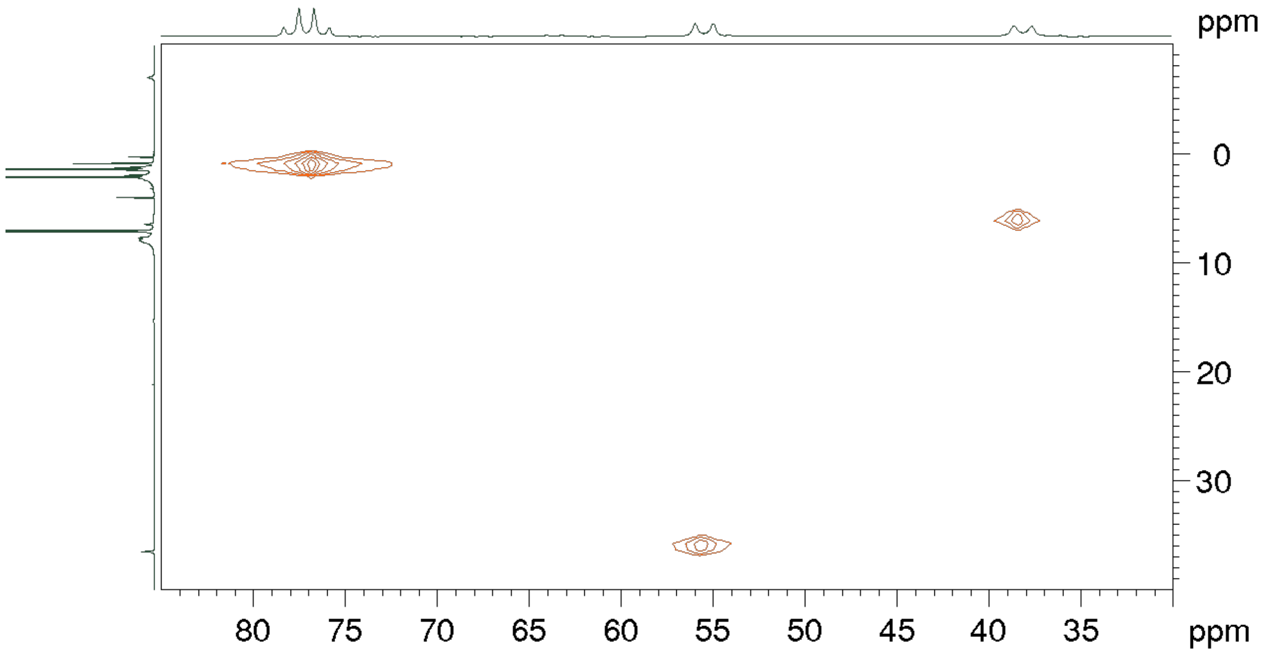

Figure S9. ^{13}C–^{1}H HETCOR NMR spectrum (150.90 MHz & 600.13 MHz, Tol-d_8, 295 K) of complex **3**.

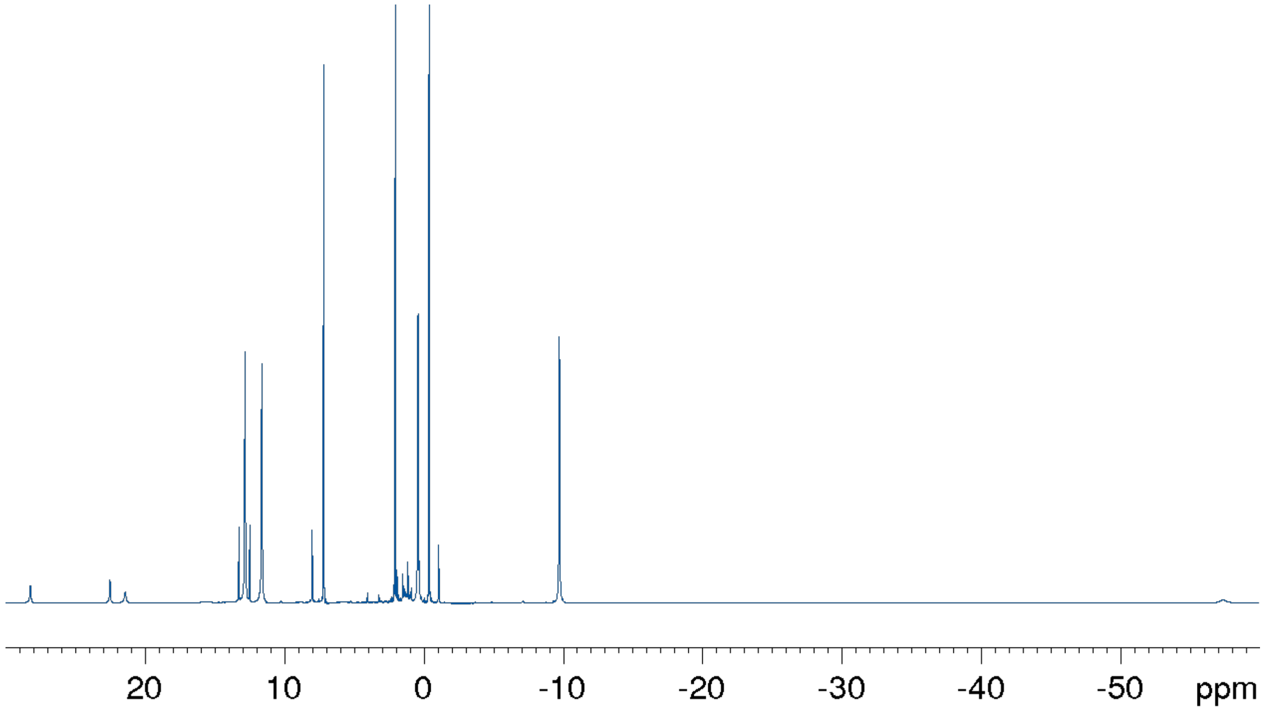

Figure S10. ^{1}H NMR spectrum (600.13 MHz, C_6D_6, 295 K) of complex **4**.

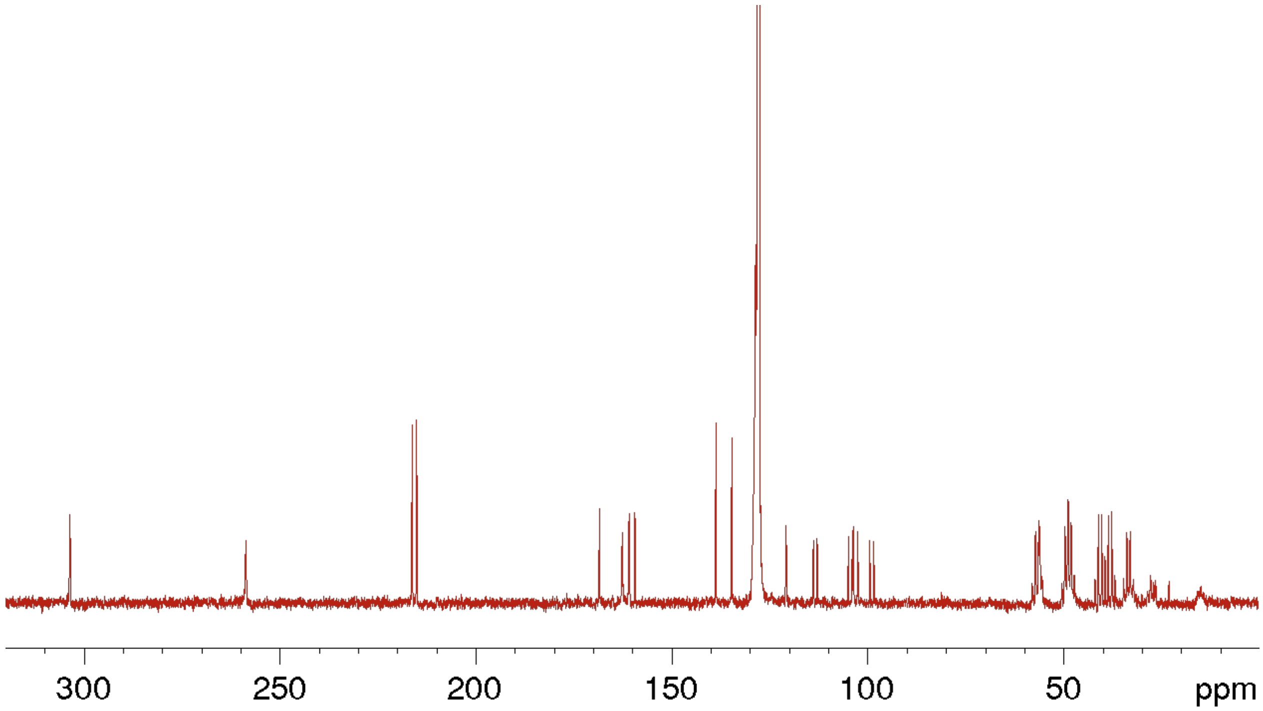

Figure S11. ^{13}C NMR spectrum (150.92 MHz, C_6D_6, 295 K) of complex **4**.

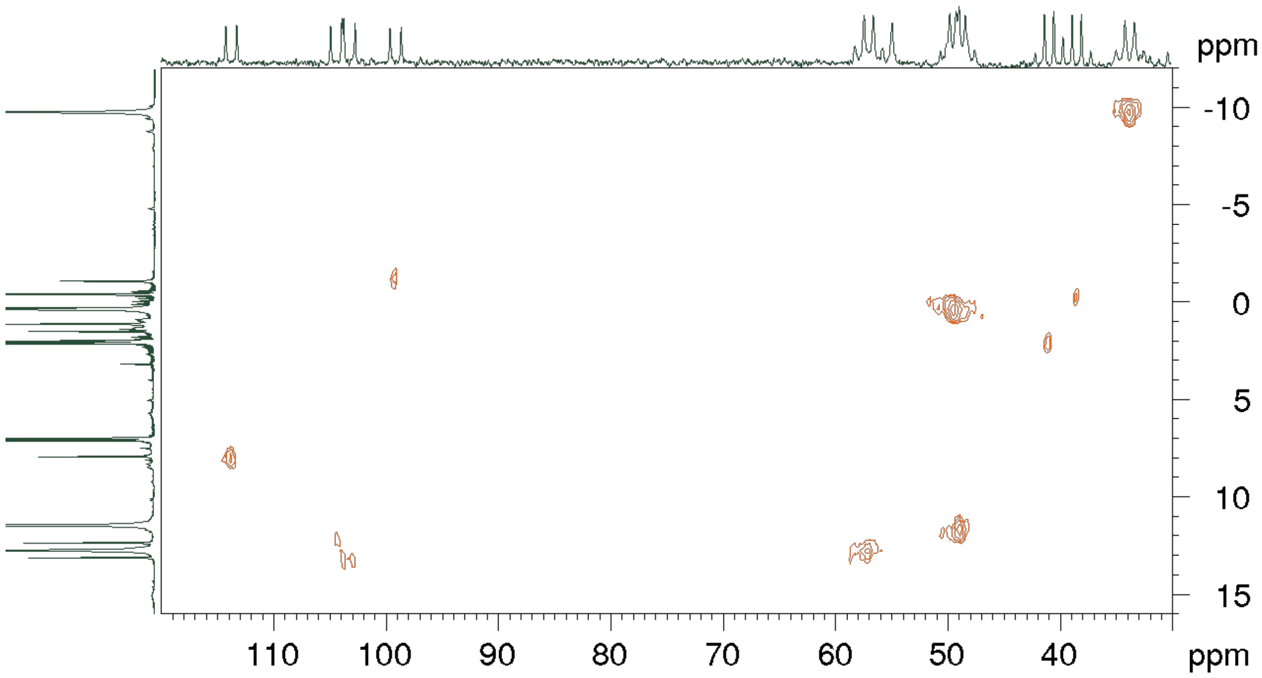

Figure S12. ^{13}C–^{1}H HETCOR NMR spectrum (150.90 MHz & 600.13 MHz, C_6D_6, 295 K) of complex **4**.

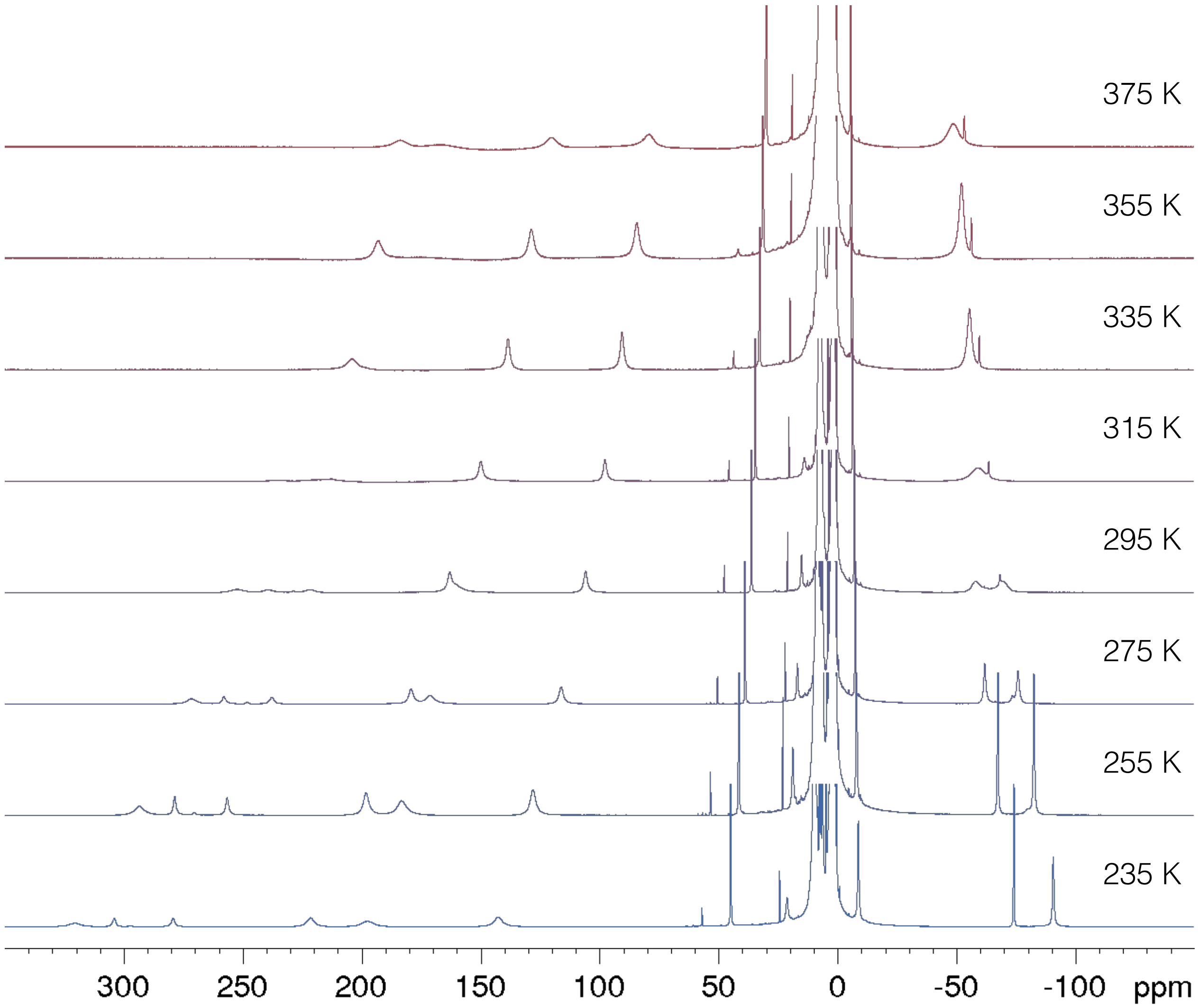

Figure S13. ^{1}H NMR spectra (399.89 MHz, Tol-d_8) of complex **3** at various temperatures.

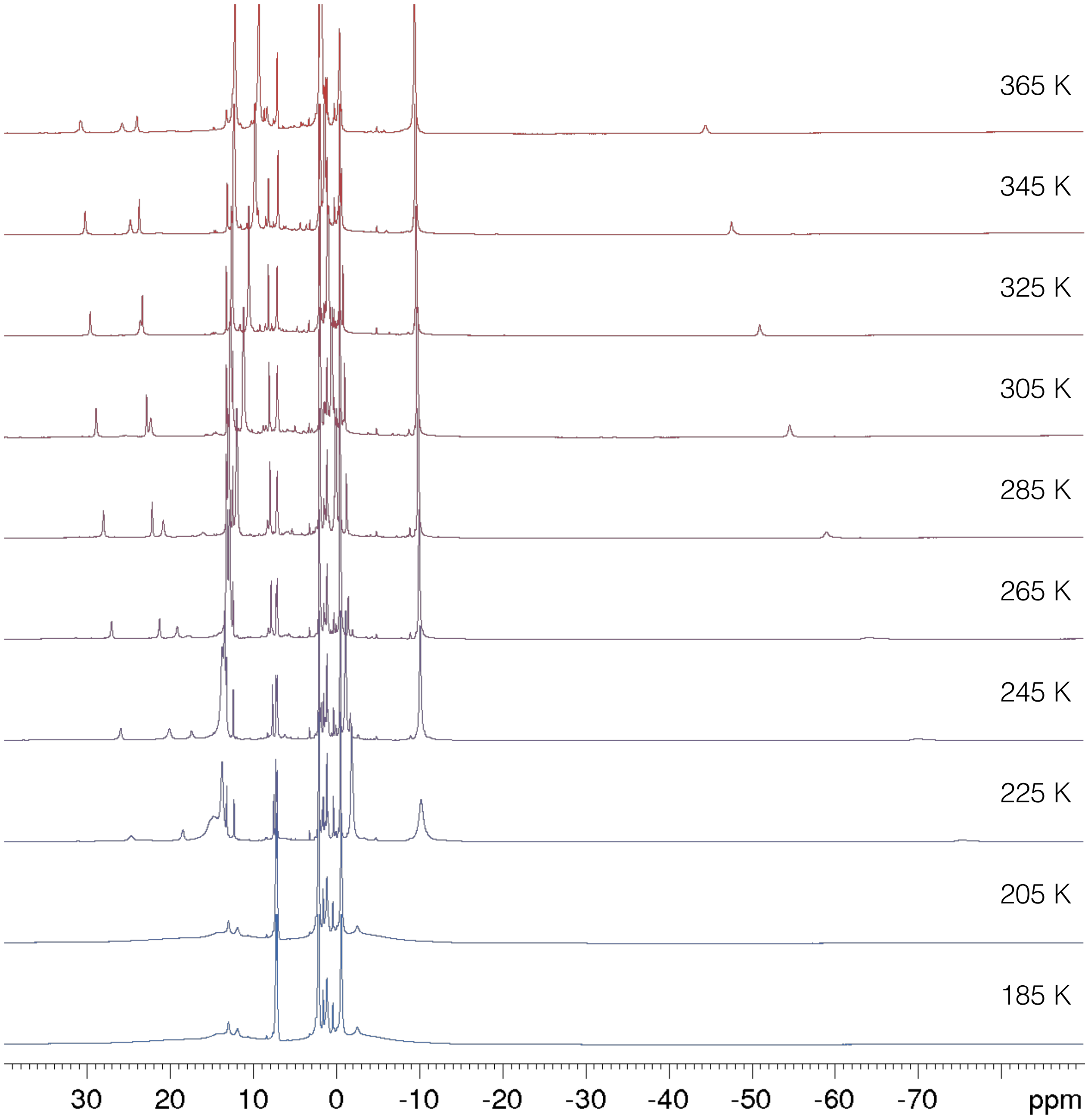

Figure S14. ^{1}H NMR spectra (399.89 MHz, Tol-d_8) of complex **4** at various temperatures.

S3 DENSITY FUNCTIONAL THEORY CALCULATIONS

S.3.1 COMPUTATIONAL DETAILS

All calculations were carried out at the density functional theory (DFT) level of theory, using the B3LYP functional along with the 6-311G(d,p) basis set for all atoms except iron, for which the def2-TZVP basis set was used. All calculations were performed with the Gaussian 09 Rev. D.01 software package.[13] Geometry optimizations for all structures were performed in the gas phase with no symmetry restrictions and the convergence criteria 'very tight'. The molecular structures of compounds **2**, **3** and **4** obtained from X-ray crystallography were used as the initial structural guesses for the geometry optimizations. Frequency calculations at the same level of theory were conducted for the freely relaxed molecules at the optimized geometries to distinguish the structures as stationary points on the potential energy surface, showing no imaginary frequencies. Furthermore, the wavefunctions generated by the self-consistent field (SCF) calculations were checked for stability by the 'stable' keyword.

Broken symmetry (BS) calculations were conducted for compound **4** in order to generate an antiferromagnetic coupled ground state, which is accounted for by the distribution of alpha and beta spin of the Mulliken spin densities ρ_{M} (Table S7, positive for alpha spin, negative for beta spin). After the antiferromagnetic guess was generated, the geometries of the structures were optimized akin to the high-spin calculations with the same convergence criteria 'very tight'. The deviation of the Mulliken spin populations from 4 electrons for each iron center is a result of the spin delocalization towards the ligands.

Table S7. Mulliken spin densities of compound **4** calculated for the nonet (9et) spin isomer and the antiferromagnetic singlet (1et) spin isomer.

#	ρ_{M} (**4**, 9et)	$\rho_{\mathrm{M,BS}}$ (**4**, 1et)
Fe(1)	3.64	–3.56
Fe(2)	3.64	3.56

The standard notation BS(n,m) for the broken symmetry solution represents the (m+n) unpaired spins of a system with spin-up and spin-down, respectively.[14] For the system investigated in this study, two antiferromagnetically coupled high-spin Fe(II) centers were established for complex **4**. The exchange coupling constant J_{AFC} was determined by Eq. S8, where E_{hs} is the energy of the nonet spin isomer, E_{BS} is the energy of the antiferromagnetically coupled singlet spin isomer and S is the total spin of the system (Table S8).[15]

$$J_{AFC} = \frac{E_{hs} - E_{BS}}{S^2} \tag{S8}$$

Table S8. Summary of the BS-DFT calculations for complex **4**.

#	BS description	E_{hs} [Hartree]	E_{BS} [Hartree]	J_{AFC} [cm^{-1}]
**4**	BS(4,4)	−7049.588	−7049.595	−99.1

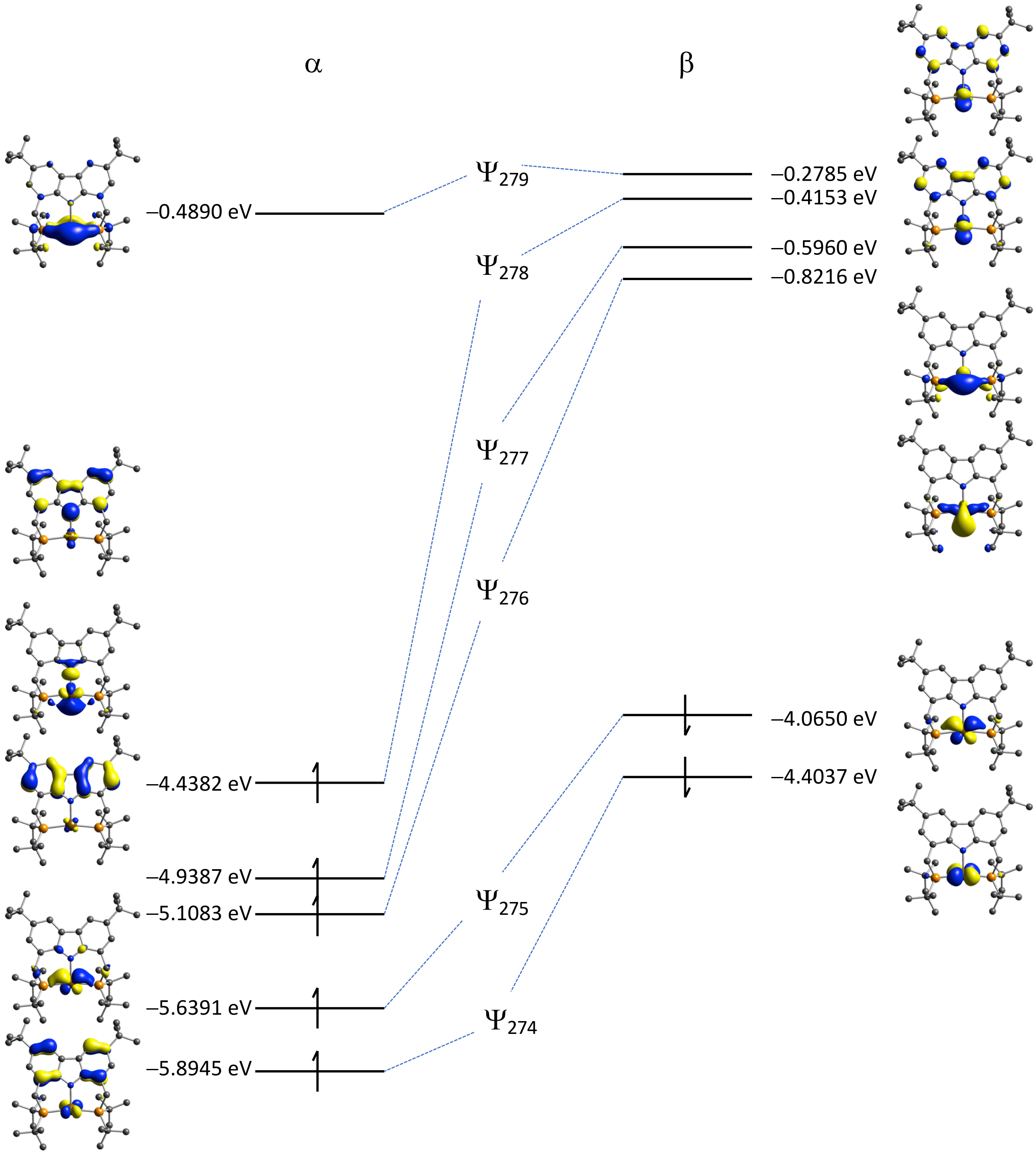

Figure S15. Spin-unrestricted Molecular Orbital (MO) energy-level diagram of complex **2** as determined by DFT (B3LYP/6-311G(d,p)//def2-tzvp(only iron)) of the frontier molecular orbitals. Alpha (left) and beta (right) MOs are depicted.

S.3.2 COORDINATES OF DFT-OPTIMIZED STRUCTURES

^{tBu}(PNP)Fe (2), 4et

Fe	-0.03403	-1.69548	-0.07329
P	-2.34292	-1.91420	-0.04858
P	2.26716	-1.98119	-0.03256
N	-0.00249	0.29539	-0.65632
C	1.11216	1.12278	-0.54881
C	2.48070	0.79586	-0.67173
C	3.41003	1.82023	-0.50935
H	4.45848	1.56215	-0.62296
C	3.06840	3.16498	-0.23938
C	1.71549	3.47706	-0.14871
H	1.39228	4.49397	0.03586
C	0.75082	2.47622	-0.30780
C	-0.68912	2.49866	-0.30741
C	-1.62408	3.52135	-0.14641
H	-1.27625	4.53221	0.03971
C	-2.99007	3.25045	-0.23527
C	-3.37227	1.92252	-0.51081
H	-4.42413	1.69033	-0.62671
C	-2.46764	0.87089	-0.67712
C	-1.09333	1.15436	-0.55069
C	-2.97413	-0.49065	-1.08597
H	-2.59809	-0.71108	-2.08914
H	-4.06547	-0.48800	-1.14092
C	2.94692	-0.58236	-1.07232
H	4.03803	-0.61316	-1.12134
H	2.56956	-0.79456	-2.07671
C	4.18579	4.21381	-0.08656
C	5.01393	4.29088	-1.39069
H	5.48043	3.33269	-1.63154
H	5.81111	5.03581	-1.29656
H	4.38007	4.57369	-2.23544
C	5.11745	3.82092	1.08363
H	4.55858	3.76709	2.02199
H	5.91682	4.55971	1.20441
H	5.58633	2.84789	0.91847
C	3.62870	5.61967	0.20399
H	2.98503	5.97542	-0.60494
H	4.45464	6.32945	0.30610
H	3.05389	5.64363	1.13382
C	-4.01462	4.38896	-0.06044
C	-3.83656	5.04071	1.33060
H	-4.00277	4.30912	2.12627
H	-4.55037	5.86029	1.46550
H	-2.83161	5.44863	1.45941
C	-5.47047	3.89783	-0.16757
H	-5.68421	3.46584	-1.14911
H	-6.15349	4.73984	-0.02313
H	-5.70365	3.14900	0.59465
C	-3.79279	5.46000	-1.15410
H	-2.78863	5.88653	-1.10153
H	-4.51172	6.27874	-1.04307
H	-3.91831	5.02767	-2.15057
C	-3.05048	-3.44663	-0.97726
C	-2.44143	-4.73120	-0.37930
H	-1.34894	-4.69608	-0.39126

H	-2.75401	-5.59301	-0.97943
H	-2.75959	-4.91317	0.64642
C	-4.58503	-3.55361	-0.97677
H	-4.98740	-3.74848	0.01879
H	-4.89166	-4.38811	-1.61798
H	-5.06247	-2.65223	-1.36827
C	-2.56600	-3.34813	-2.44069
H	-3.07092	-2.55675	-2.99666
H	-2.77878	-4.29318	-2.95169
H	-1.48741	-3.17534	-2.50596
C	-3.20022	-1.69243	1.66037
C	-3.20537	-3.01137	2.45392
H	-3.91192	-3.73982	2.05255
H	-3.50720	-2.80221	3.48614
H	-2.21471	-3.47284	2.48936
C	-4.63376	-1.13957	1.56921
H	-4.65257	-0.14321	1.12599
H	-5.04818	-1.05333	2.58018
H	-5.30053	-1.78571	0.99724
C	-2.33061	-0.67693	2.43440
H	-1.32505	-1.06740	2.61405
H	-2.78920	-0.48261	3.41064
H	-2.23805	0.27403	1.90914
C	2.93499	-3.53817	-0.94996
C	2.46558	-3.43017	-2.41763
H	1.39423	-3.22012	-2.49211
H	2.64918	-4.38441	-2.92279
H	3.00230	-2.65958	-2.97301
C	2.28054	-4.79981	-0.35140
H	2.58546	-4.98768	0.67735
H	2.56890	-5.67382	-0.94592
H	1.18993	-4.72862	-0.37094
C	4.46528	-3.69383	-0.93660
H	4.97444	-2.81168	-1.33233
H	4.75004	-4.54330	-1.56816
H	4.85356	-3.89244	0.06380
C	3.12132	-1.77713	1.68039
C	3.07977	-3.09196	2.47966
H	2.07409	-3.51988	2.51323
H	3.38453	-2.88840	3.51214
H	3.76300	-3.84542	2.08411
C	4.57221	-1.27047	1.59508
H	5.22162	-1.94068	1.03090
H	4.98290	-1.19144	2.60814
H	4.62519	-0.27775	1.14665
C	2.27975	-0.73140	2.44520
H	2.21984	0.22007	1.91617
H	2.73901	-0.54746	3.42309
H	1.26141	-1.08921	2.62079

^{tBu}(PNP)Fe(OCPh$_2$) (3), 4et

Fe	-0.91018	0.17260	-0.49287
P	-1.05230	-1.98142	-1.88188
P	-0.87152	2.59527	-1.02784
O	-2.30346	-0.00191	0.78421
N	1.10344	-0.04952	-0.46907
C	2.06197	0.94866	-0.34846
C	1.99995	2.29939	-0.75653

C	3.10780	3.09933	-0.48728	C	4.95215	4.77142	1.30032
H	3.06399	4.13489	-0.80986	H	4.13300	5.32606	0.83689
C	4.29002	2.63336	0.13418	H	5.76412	5.47910	1.49763
C	4.36292	1.28005	0.44717	H	4.59405	4.38761	2.25953
H	5.26083	0.85820	0.88034	C	4.33770	-4.53947	1.09284
C	3.27147	0.44115	0.19536	C	3.66949	-5.28826	2.26942
C	3.07204	-0.97950	0.33666	H	3.55254	-4.62735	3.13259
C	3.91515	-2.02306	0.73189	H	4.27818	-6.14538	2.57586
H	4.92224	-1.79274	1.05579	H	2.67869	-5.66170	2.00052
C	3.46512	-3.33889	0.68178	C	5.74593	-4.10869	1.54289
C	2.14763	-3.56468	0.22413	H	6.28850	-3.59671	0.74347
H	1.78494	-4.58633	0.17034	H	6.32739	-4.99066	1.82576
C	1.26584	-2.55586	-0.16217	H	5.70905	-3.44469	2.41069
C	1.73992	-1.22795	-0.09378	C	4.49640	-5.50776	-0.10277
C	-0.14733	-2.95362	-0.55234	H	3.53342	-5.89981	-0.43849
H	-0.79362	-2.84183	0.32402	H	5.12347	-6.36110	0.17540
H	-0.16007	-4.01708	-0.80519	H	4.96553	-5.00235	-0.95136
C	0.88254	2.84360	-1.62233	C	-2.74447	-2.89746	-1.95897
H	0.91875	2.31577	-2.57963	C	-3.73478	-1.99566	-2.72576
H	1.06203	3.89951	-1.83632	H	-3.83374	-1.02323	-2.23819
C	5.44582	3.61683	0.39744	H	-4.72336	-2.46717	-2.72853
C	6.63523	2.94003	1.10270	H	-3.44617	-1.83060	-3.76403
H	6.34476	2.51742	2.06829	C	-2.70640	-4.29844	-2.59529
H	7.42278	3.67653	1.28511	H	-2.45365	-4.27959	-3.65556
H	7.06564	2.14008	0.49442	H	-3.69987	-4.75249	-2.50709
C	5.95425	4.19959	-0.94227	H	-2.00393	-4.96217	-2.08532
H	6.31681	3.40312	-1.59788	C	-3.27699	-3.04454	-0.51709
H	6.77774	4.90022	-0.76919	H	-2.71721	-3.78420	0.05837
H	5.16657	4.73706	-1.47561	H	-4.31527	-3.38893	-0.56467

H	-3.26832	-2.09850	0.02307	H	-0.92207	2.08804	1.90491
C	-0.05306	-2.28058	-3.50122	H	-0.42832	3.64724	2.56336
C	0.53630	-3.70071	-3.59418	H	0.66137	2.76254	1.49297
H	1.23480	-3.89936	-2.77948	C	-0.28736	5.10442	0.31236
H	1.09766	-3.78992	-4.53116	H	0.78718	4.96242	0.18541
H	-0.22553	-4.47979	-3.59561	H	-0.42770	5.70284	1.21925
C	1.11621	-1.27396	-3.52990	H	-0.66143	5.69181	-0.52644
H	0.76586	-0.24183	-3.48154	C	-2.50288	4.02471	0.82805
H	1.66082	-1.40313	-4.47194	H	-2.99622	4.66328	0.09264
H	1.81746	-1.42805	-2.71157	H	-2.55537	4.54074	1.79266
C	-0.93570	-1.99623	-4.73198	H	-3.07142	3.09704	0.92369
H	-1.73600	-2.72506	-4.86212	C	-2.87462	-0.14192	1.94463
H	-0.31015	-2.03661	-5.63008	C	-4.33958	-0.17208	1.96460
H	-1.38246	-0.99927	-4.69079	C	-5.05931	0.39711	0.89040
C	-1.92936	3.22061	-2.50565	H	-4.50521	0.87376	0.09261
C	-3.39393	2.80335	-2.26016	C	-6.44627	0.35846	0.85125
H	-3.47708	1.72301	-2.11949	H	-6.97168	0.81709	0.02006
H	-3.99810	3.07502	-3.13277	C	-7.16677	-0.26443	1.87160
H	-3.83205	3.29195	-1.39044	H	-8.24983	-0.29636	1.83940
C	-1.44227	2.47680	-3.76724	C	-6.47335	-0.85620	2.92828
H	-0.46887	2.83057	-4.11198	H	-7.01854	-1.36667	3.71502
H	-2.15758	2.64874	-4.57836	C	-5.08575	-0.81377	2.97775
H	-1.38056	1.39778	-3.60295	H	-4.57061	-1.31248	3.78822
C	-1.85490	4.73396	-2.76805	C	-2.04013	-0.25699	3.14434
H	-2.28942	5.31794	-1.95558	C	-2.48997	0.13902	4.42380
H	-2.41989	4.97136	-3.67671	H	-3.48600	0.54624	4.53849
H	-0.82897	5.07517	-2.92665	C	-1.65846	0.05871	5.53324
C	-1.02412	3.76409	0.48782	H	-2.02808	0.38234	6.50042
C	-0.38577	3.00851	1.67422	C	-0.35222	-0.41685	5.40925

H	0.29350	-0.47976	6.27771
C	0.11685	-0.79610	4.15146
H	1.13250	-1.15684	4.03373
C	-0.70779	-0.71073	3.03755
H	-0.31727	-0.99876	2.07049

(^{tBu}(PNP)Fe)$_2$O (4), 9et

Fe	-1.53184	0.98558	1.00984
P	-2.96909	3.09699	1.10900
P	-1.02020	-0.60560	3.09764
N	-3.15607	-0.24569	0.76853
C	-3.02737	-1.61713	0.57893
C	-2.04630	-2.49085	1.09353
C	-2.19365	-3.84801	0.80607
H	-1.44244	-4.52181	1.20543
C	-3.25428	-4.38941	0.04290
C	-4.22166	-3.51091	-0.43563
H	-5.07143	-3.87392	-0.99974
C	-4.12268	-2.14349	-0.15979
C	-4.99983	-1.02704	-0.40836
C	-6.24200	-0.90670	-1.04324
H	-6.66864	-1.77242	-1.53398
C	-6.92432	0.30573	-1.01955
C	-6.34293	1.37173	-0.29306
H	-6.90521	2.29387	-0.18472
C	-5.10992	1.29399	0.34874
C	-4.39058	0.08589	0.22983
C	-4.68546	2.38381	1.30712
H	-4.67701	1.94956	2.31057
H	-5.42948	3.18322	1.31466
C	-0.83895	-2.04406	1.89762
H	-0.06200	-1.69809	1.20881
H	-0.41796	-2.90804	2.41765
C	-3.31564	-5.90918	-0.20192
C	-3.37880	-6.65590	1.15118
H	-2.50194	-6.45028	1.76899
H	-3.42642	-7.73805	0.99079
H	-4.26453	-6.35585	1.71786
C	-2.05766	-6.36576	-0.97586
H	-1.99823	-5.86591	-1.94679
H	-2.08331	-7.44647	-1.15015
H	-1.14199	-6.13897	-0.42557
C	-4.55127	-6.31789	-1.02502
H	-5.48265	-6.05375	-0.51697
H	-4.55161	-7.40125	-1.17448
H	-4.55508	-5.84765	-2.01218
C	-8.29261	0.51066	-1.69605
C	-9.35809	0.86335	-0.63149
H	-9.44692	0.06168	0.10669
H	-10.33708	1.00609	-1.10078
H	-9.10942	1.78274	-0.09614
C	-8.76874	-0.74950	-2.44154
H	-8.06719	-1.04533	-3.22629
H	-9.73457	-0.55405	-2.91597
H	-8.90022	-1.59693	-1.76341
C	-8.19829	1.66364	-2.72242
H	-7.90540	2.60361	-2.24877
H	-9.16472	1.82321	-3.21185

H	-7.45848	1.43542	-3.49477	H	0.68047	1.67106	3.94309
C	-2.84474	4.28296	2.61823	H	1.89397	0.93081	4.98998
C	-1.38571	4.76629	2.74046	H	0.19578	0.90953	5.46302
H	-0.69042	3.92430	2.77888	C	1.74689	-0.49330	2.69043
H	-1.27164	5.33957	3.66725	H	1.87215	-1.47455	2.23135
H	-1.08603	5.41289	1.91654	H	2.72874	-0.19032	3.06797
C	-3.79395	5.49163	2.57212	H	1.45310	0.21252	1.91153
H	-3.54665	6.17919	1.76246	C	1.12857	-1.67448	4.79793
H	-3.71350	6.05111	3.51113	H	0.54501	-1.69286	5.71941
H	-4.83895	5.19268	2.46085	H	2.18131	-1.56431	5.08190
C	-3.16152	3.45150	3.87975	H	1.02963	-2.64560	4.30767
H	-4.21943	3.19424	3.95627	C	-2.27530	-1.22469	4.42983
H	-2.90441	4.04100	4.76620	C	-3.70207	-0.96312	3.90429
H	-2.57627	2.52906	3.91397	H	-3.85809	0.08992	3.66625
C	-3.07646	4.08414	-0.53625	H	-4.41748	-1.24420	4.68572
C	-2.82948	3.05029	-1.65489	H	-3.93187	-1.54494	3.01365
H	-1.84983	2.58105	-1.54623	C	-2.14412	-2.72968	4.73081
H	-2.86505	3.56013	-2.62464	H	-2.32366	-3.33464	3.84046
H	-3.59002	2.26803	-1.66291	H	-2.90152	-3.01010	5.47208
C	-1.96060	5.13934	-0.61364	H	-1.17111	-2.99890	5.14122
H	-2.11478	5.96866	0.07891	C	-2.11524	-0.41739	5.73251
H	-1.94043	5.55934	-1.62524	H	-1.16988	-0.60857	6.23995
H	-0.98120	4.69849	-0.42246	H	-2.91815	-0.70073	6.42167
C	-4.43912	4.75935	-0.77588	H	-2.20066	0.65869	5.55950
H	-5.24746	4.03015	-0.83789	Fe	1.53184	0.98558	-1.00984
H	-4.40578	5.28551	-1.73677	P	2.96909	3.09699	-1.10900
H	-4.69220	5.49525	-0.01148	P	1.02020	-0.60560	-3.09764
C	0.74446	-0.51550	3.86156	O	0.00000	1.26263	0.00000
C	0.87129	0.82787	4.61107	N	3.15607	-0.24568	-0.76853

C	3.02737	-1.61713	-0.57893	H	2.08331	-7.44647	1.15014
C	2.04629	-2.49085	-1.09353	H	1.14199	-6.13897	0.42557
C	2.19365	-3.84801	-0.80607	C	4.55127	-6.31789	1.02501
H	1.44244	-4.52181	-1.20544	H	5.48265	-6.05375	0.51695
C	3.25428	-4.38941	-0.04291	H	4.55162	-7.40125	1.17446
C	4.22166	-3.51091	0.43562	H	4.55508	-5.84765	2.01216
H	5.07143	-3.87392	0.99973	C	8.29261	0.51065	1.69606
C	4.12268	-2.14350	0.15978	C	9.35809	0.86334	0.63149
C	4.99983	-1.02705	0.40836	H	9.44692	0.06168	-0.10669
C	6.24200	-0.90670	1.04324	H	10.33708	1.00609	1.10078
H	6.66863	-1.77242	1.53397	H	9.10942	1.78274	0.09615
C	6.92432	0.30573	1.01955	C	8.76874	-0.74950	2.44154
C	6.34293	1.37173	0.29307	H	8.06719	-1.04533	3.22629
H	6.90521	2.29387	0.18473	H	9.73456	-0.55406	2.91597
C	5.10992	1.29399	-0.34873	H	8.90021	-1.59693	1.76341
C	4.39058	0.08589	-0.22983	C	8.19829	1.66363	2.72243
C	4.68546	2.38381	-1.30712	H	7.90540	2.60361	2.24877
H	4.67701	1.94956	-2.31056	H	9.16472	1.82320	3.21186
H	5.42948	3.18322	-1.31465	H	7.45848	1.43542	3.49477
C	0.83895	-2.04406	-1.89763	C	2.84475	4.28296	-2.61822
H	0.06199	-1.69809	-1.20882	C	1.38572	4.76630	-2.74045
H	0.41796	-2.90803	-2.41766	H	0.69042	3.92431	-2.77887
C	3.31564	-5.90918	0.20191	H	1.27164	5.33958	-3.66724
C	3.37879	-6.65590	-1.15119	H	1.08604	5.41290	-1.91653
H	2.50193	-6.45028	-1.76900	C	3.79396	5.49164	-2.57211
H	3.42641	-7.73805	-0.99081	H	3.54666	6.17919	-1.76245
H	4.26452	-6.35585	-1.71788	H	3.71351	6.05112	-3.51112
C	2.05766	-6.36577	0.97585	H	4.83896	5.19268	-2.46084
H	1.99823	-5.86592	1.94678	C	3.16152	3.45150	-3.87975

H	4.21943	3.19425	-3.95627
H	2.90442	4.04101	-4.76620
H	2.57626	2.52907	-3.91397
C	3.07646	4.08414	0.53626
C	2.82947	3.05029	1.65490
H	1.84982	2.58105	1.54624
H	2.86504	3.56013	2.62464
H	3.59001	2.26803	1.66291
C	1.96060	5.13934	0.61365
H	2.11478	5.96867	-0.07890
H	1.94043	5.55934	1.62525
H	0.98120	4.69849	0.42247
C	4.43912	4.75935	0.77589
H	5.24746	4.03015	0.83790
H	4.40578	5.28550	1.73678
H	4.69221	5.49525	0.01149
C	-0.74446	-0.51549	-3.86156
C	-0.87129	0.82788	-4.61107
H	-0.68047	1.67107	-3.94308
H	-1.89397	0.93082	-4.98997
H	-0.19578	0.90954	-5.46302
C	-1.74689	-0.49330	-2.69044
H	-1.87215	-1.47455	-2.23135
H	-2.72874	-0.19032	-3.06797
H	-1.45310	0.21253	-1.91153
C	-1.12857	-1.67447	-4.79794
H	-0.54501	-1.69285	-5.71941
H	-2.18131	-1.56429	-5.08190
H	-1.02963	-2.64559	-4.30767
C	2.27530	-1.22468	-4.42983
C	3.70207	-0.96311	-3.90429
H	3.85809	0.08993	-3.66625
H	4.41748	-1.24419	-4.68572
H	3.93187	-1.54494	-3.01365
C	2.14412	-2.72967	-4.73082
H	2.32366	-3.33463	-3.84047
H	2.90152	-3.01009	-5.47208
H	1.17111	-2.99889	-5.14122
C	2.11524	-0.41738	-5.73252
H	1.16988	-0.60856	-6.23995
H	2.91816	-0.70071	-6.42167
H	2.20066	0.65870	-5.55950

(^{tBu}(PNP)Fe)$_2$O (4), BS-1et

Fe	1.49720	0.98965	-1.01033
P	2.93641	3.09561	-1.11307
P	0.98806	-0.60236	-3.09982
N	3.12123	-0.24552	-0.76906
C	2.99383	-1.61702	-0.58103
C	2.01362	-2.49080	-1.09717
C	2.16282	-3.84858	-0.81317
H	1.41221	-4.52236	-1.21369
C	3.22524	-4.39061	-0.05273
C	4.19222	-3.51217	0.42666
H	5.04381	-3.87574	0.98768
C	4.09114	-2.14420	0.15458
C	4.96859	-1.02790	0.40211
C	6.21322	-0.90744	1.03232
H	6.64142	-1.77284	1.52229

C	6.89630	0.30449	1.00425	C	8.74449	-0.74958	2.42225
C	6.31360	1.36954	0.27718	H	8.04498	-1.04404	3.20934
H	6.87707	2.29034	0.16382	H	9.71168	-0.55382	2.89378
C	5.07790	1.29161	-0.35930	H	8.87379	-1.59803	1.74498
C	4.35730	0.08499	-0.23419	C	8.17585	1.66416	2.70135
C	4.64967	2.37720	-1.32044	H	7.88190	2.60355	2.22718
H	4.63305	1.93732	-2.32128	H	9.14372	1.82410	3.18783
H	5.39524	3.17490	-1.33763	H	7.43817	1.43730	3.47613
C	0.80702	-2.04342	-1.90234	C	2.80776	4.27498	-2.62755
H	0.02747	-1.70164	-1.21457	C	1.35377	4.77569	-2.73706
H	0.38892	-2.90672	-2.42572	H	0.64696	3.94275	-2.75744
C	3.28921	-5.91082	0.18882	H	1.23556	5.34019	-3.66870
C	3.34117	-6.65575	-1.16573	H	1.07329	5.43555	-1.91695
H	2.45855	-6.45099	-1.77553	C	3.77160	5.47259	-2.60177
H	3.39178	-7.73801	-1.00702	H	3.54148	6.16916	-1.79479
H	4.22138	-6.35383	-1.73999	H	3.68657	6.02587	-3.54405
C	2.03799	-6.36868	0.97281	H	4.81440	5.16265	-2.50048
H	1.98789	-5.87153	1.94566	C	3.09957	3.43084	-3.88667
H	2.06405	-7.44991	1.14377	H	4.15334	3.16035	-3.97396
H	1.11775	-6.13913	0.43150	H	2.83877	4.01671	-4.77446
C	4.53184	-6.32071	1.00072	H	2.50322	2.51509	-3.90661
H	5.45880	-6.05421	0.48588	C	3.06488	4.09177	0.52603
H	4.53425	-7.40449	1.14716	C	2.82232	3.06596	1.65298
H	4.54324	-5.85344	1.98922	H	1.83908	2.60183	1.55672
C	8.26676	0.50970	1.67629	H	2.87055	3.58077	2.61954
C	9.32944	0.86047	0.60835	H	3.57773	2.27876	1.65766
H	9.41590	0.05774	-0.12895	C	1.95814	5.15604	0.60838
H	10.30976	1.00346	1.07480	H	2.11368	5.98154	-0.08848
H	9.07976	1.77925	0.07241	H	1.94939	5.58007	1.61845

H	0.97347	4.72326	0.42661	H	2.89631	-0.69332	-6.41874
C	4.43402	4.75968	0.74904	H	2.17232	0.66471	-5.55997
H	5.23816	4.02592	0.81030	Fe	-1.49720	0.98965	1.01033
H	4.41200	5.29398	1.70576	P	-2.93641	3.09561	1.11307
H	4.68521	5.48750	-0.02366	P	-0.98806	-0.60236	3.09981
C	-0.77393	-0.51934	-3.87169	O	0.00000	1.26286	0.00000
C	-0.90333	0.82195	-4.62436	N	-3.12123	-0.24552	0.76906
H	-0.71838	1.66727	-3.95749	C	-2.99382	-1.61702	0.58103
H	-1.92508	0.92001	-5.00707	C	-2.01362	-2.49080	1.09717
H	-0.22523	0.90477	-5.47406	C	-2.16282	-3.84858	0.81317
C	-1.78162	-0.49862	-2.70515	H	-1.41221	-4.52236	1.21369
H	-1.90328	-1.47825	-2.24195	C	-3.22524	-4.39061	0.05273
H	-2.76356	-0.20283	-3.08804	C	-4.19221	-3.51217	-0.42666
H	-1.49694	0.21249	-1.92809	H	-5.04381	-3.87574	-0.98767
C	-1.15055	-1.68142	-4.80726	C	-4.09114	-2.14420	-0.15458
H	-0.56377	-1.69908	-5.72665	C	-4.96859	-1.02790	-0.40211
H	-2.20269	-1.57572	-5.09512	C	-6.21322	-0.90745	-1.03232
H	-1.04956	-2.65133	-4.31505	H	-6.64142	-1.77284	-1.52229
C	2.24974	-1.21758	-4.42833	C	-6.89630	0.30449	-1.00424
C	3.67458	-0.95164	-3.89990	C	-6.31360	1.36953	-0.27718
H	3.82682	0.10171	-3.66121	H	-6.87707	2.29034	-0.16382
H	4.39220	-1.23003	-4.68027	C	-5.07790	1.29161	0.35930
H	3.90479	-1.53304	-3.00915	C	-4.35730	0.08499	0.23419
C	2.12460	-2.72322	-4.72921	C	-4.64967	2.37720	1.32044
H	2.30352	-3.32737	-3.83822	H	-4.63305	1.93732	2.32128
H	2.88549	-3.00089	-5.46792	H	-5.39524	3.17490	1.33763
H	1.15408	-2.99623	-5.14293	C	-0.80702	-2.04342	1.90234
C	2.09064	-0.41176	-5.73209	H	-0.02747	-1.70164	1.21457
H	1.14735	-0.60646	-6.24200	H	-0.38891	-2.90672	2.42572

C	-3.28921	-5.91083	-0.18882	H	-1.23556	5.34019	3.66870
C	-3.34117	-6.65575	1.16574	H	-1.07330	5.43556	1.91695
H	-2.45854	-6.45099	1.77553	C	-3.77161	5.47258	2.60177
H	-3.39178	-7.73801	1.00702	H	-3.54149	6.16915	1.79479
H	-4.22137	-6.35383	1.73999	H	-3.68658	6.02587	3.54405
C	-2.03800	-6.36868	-0.97281	H	-4.81441	5.16264	2.50049
H	-1.98789	-5.87153	-1.94566	C	-3.09957	3.43084	3.88667
H	-2.06405	-7.44991	-1.14378	H	-4.15334	3.16034	3.97396
H	-1.11774	-6.13913	-0.43150	H	-2.83877	4.01671	4.77446
C	-4.53184	-6.32071	-1.00072	H	-2.50321	2.51509	3.90661
H	-5.45880	-6.05421	-0.48587	C	-3.06488	4.09177	-0.52603
H	-4.53425	-7.40449	-1.14715	C	-2.82232	3.06597	-1.65298
H	-4.54325	-5.85344	-1.98922	H	-1.83908	2.60184	-1.55672
C	-8.26676	0.50970	-1.67629	H	-2.87055	3.58077	-2.61954
C	-9.32944	0.86047	-0.60834	H	-3.57773	2.27876	-1.65766
H	-9.41590	0.05774	0.12896	C	-1.95815	5.15605	-0.60838
H	-10.30976	1.00346	-1.07479	H	-2.11369	5.98154	0.08848
H	-9.07976	1.77925	-0.07241	H	-1.94939	5.58007	-1.61845
C	-8.74449	-0.74958	-2.42225	H	-0.97347	4.72326	-0.42661
H	-8.04498	-1.04404	-3.20933	C	-4.43403	4.75967	-0.74904
H	-9.71168	-0.55382	-2.89377	H	-5.23816	4.02592	-0.81030
H	-8.87379	-1.59803	-1.74498	H	-4.41200	5.29397	-1.70576
C	-8.17585	1.66415	-2.70134	H	-4.68522	5.48749	0.02366
H	-7.88190	2.60355	-2.22718	C	0.77393	-0.51934	3.87169
H	-9.14372	1.82409	-3.18783	C	0.90333	0.82195	4.62436
H	-7.43817	1.43730	-3.47613	H	0.71838	1.66727	3.95749
C	-2.80776	4.27498	2.62755	H	1.92508	0.92001	5.00707
C	-1.35377	4.77569	2.73706	H	0.22523	0.90477	5.47406
H	-0.64696	3.94275	2.75744	C	1.78163	-0.49862	2.70515

H	1.90328	-1.47825	2.24195
H	2.76357	-0.20283	3.08804
H	1.49694	0.21249	1.92809
C	1.15055	-1.68142	4.80726
H	0.56377	-1.69908	5.72665
H	2.20269	-1.57572	5.09512
H	1.04956	-2.65133	4.31505
C	-2.24974	-1.21758	4.42833
C	-3.67458	-0.95164	3.89990
H	-3.82682	0.10171	3.66121
H	-4.39220	-1.23003	4.68027
H	-3.90479	-1.53304	3.00915
C	-2.12459	-2.72322	4.72921
H	-2.30352	-3.32737	3.83821
H	-2.88549	-3.00089	5.46792
H	-1.15407	-2.99623	5.14293
C	-2.09063	-0.41176	5.73209
H	-1.14735	-0.60646	6.24200
H	-2.89631	-0.69332	6.41874
H	-2.17232	0.66471	5.55997

S4 SQUID Magnetometry

S4.1 General Procedure

SQUID measurements were carried out in polycarbonate capsules (Quantum Design, QDS-AGC3). The diamagnetic contribution of the sample was calculated with the aid of Pascal constants and together with the background measured for the capsule was subtracted from the data. A Quantum Design MPMS XL SQUID magnetometer was used for the measurements. The solid material was powdered (*agate mortar*) before being transferred to the capsules. The filled capsules were then rapidly transferred from an argon flooded Schlenk flask to the SQUID magnetometer and evacuated immediately. However, due to the high sensitivity towards oxygen and moisture of compound **4** the comparably high paramagnetic impurities observed in the measurements (Figure S16) were assigned to a reaction product of the compounds with atmospheric oxygen formed during the transfer into the magnetometer. The fit of the data was performed with the software package PHI[16] assuming two coupled S = 2 centers.

S4.2 Magnetometry Data

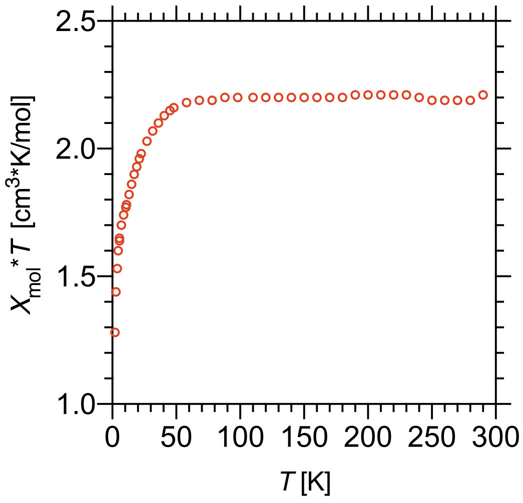

Figure S16. Variable temperature magnetic susceptibility data for ^{tBu}(PNP)Fe (**2**) recorded at an external magnetic field of 1.0 T across the temperature range from 2 K to 290 K (red cycles).

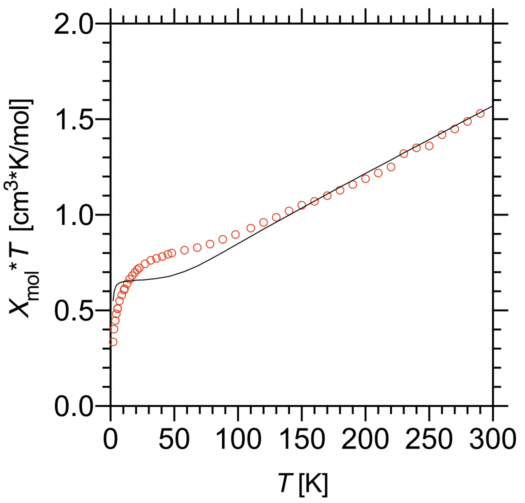

Figure S17. Variable temperature magnetic susceptibility data for (^{tBu}(PNP)Fe)$_2$O (**4**) recorded at an external magnetic field of 1.0 T across the temperature range from 2 K to 290 K (red cycles). The data was fitted using the software package PHI[16] (black line) assuming two coupled S = 2 centers with the inclusion of 21 % of an S = 2 impurity and a TIP of 5×10^{-4} cm^3/mol with the following parameters: $g = 2.00$; $J = -87$ cm^{-1}.

S5 Absorption Spectra

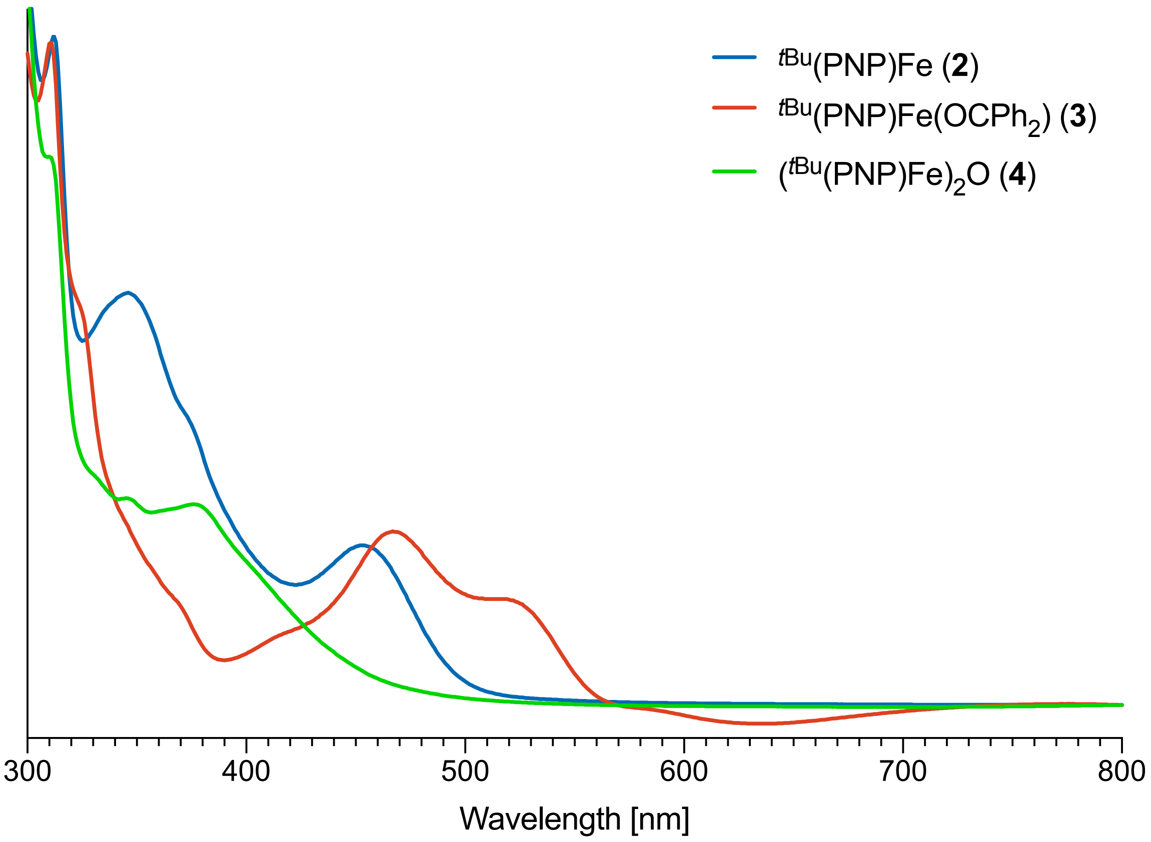

Figure S18. Normalized absorption spectra of complexes **2**, **3** and **4** recorded in *n*-hexane.

S6 EPR Spectra

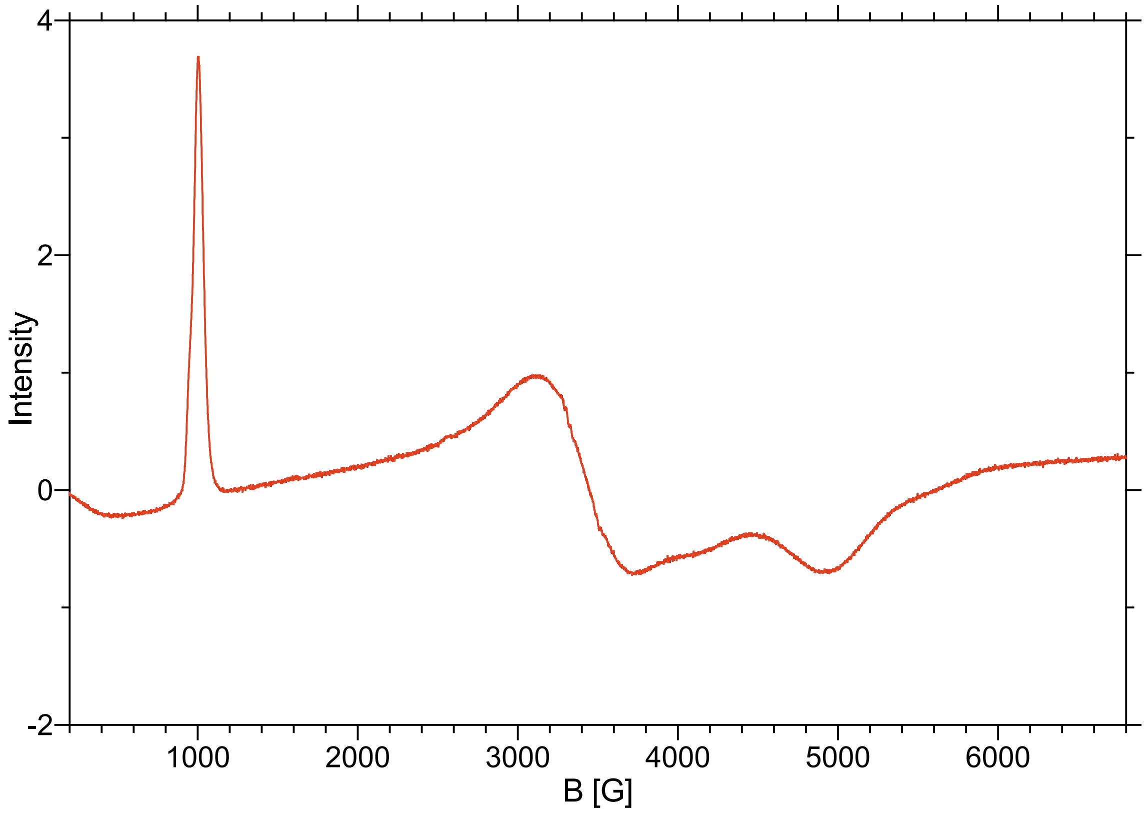

Figure S19. X-band EPR spectrum of complex **2** at 5.9 K in toluene glass (microwave fifrequency 9.636898 MHz).

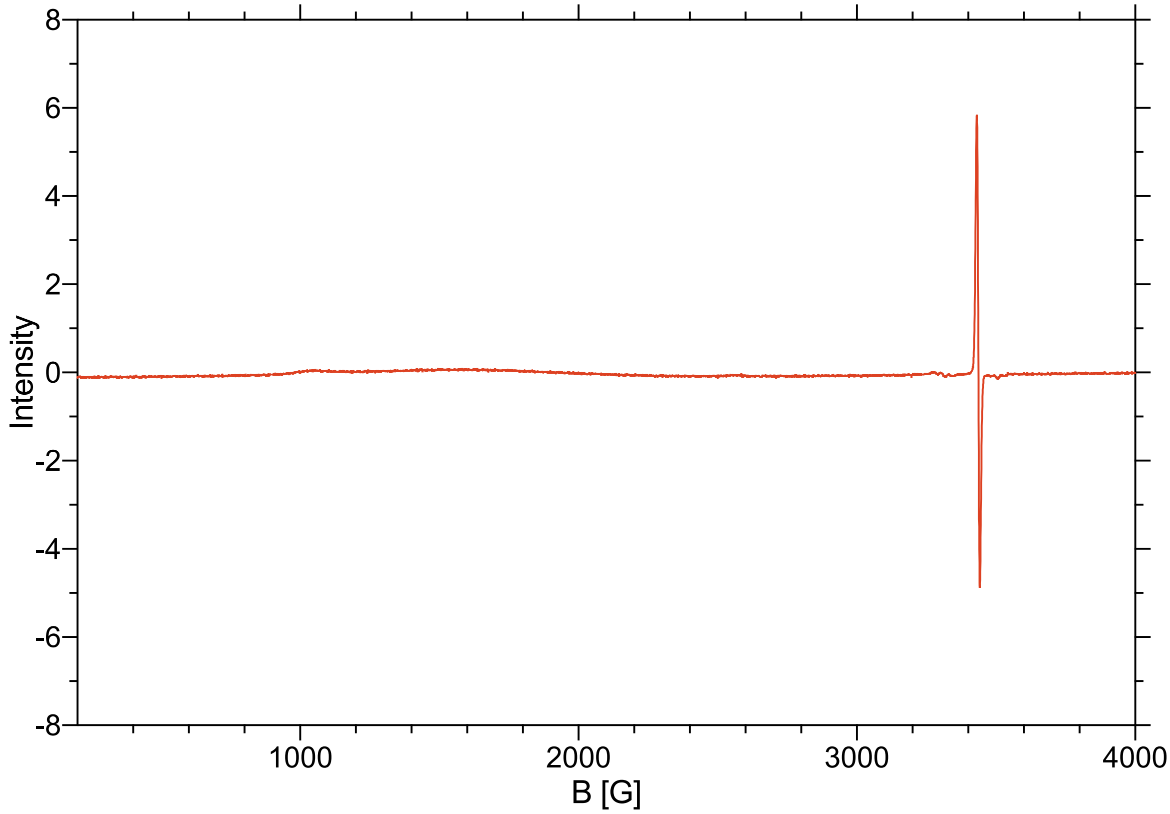

Figure S20. X-band EPR spectrum of complex **3** at 6.0 K in toluene glass (microwave frequency 9.637376 MHz).

S7 Crystallographic Data

S7.1 X-ray Crystal Structure Determinations

Crystal data and details of the structure determinations are compiled in Table S9. Full shells of intensity data were collected at low temperature with an Agilent Technologies Supernova-E CCD diffractometer (Cu-K_α radiation, microfocus X-ray tube, multilayer mirror optics). Detector frames (typically ω-, occasionally φ-scans, scan width 1°) were integrated by profile fitting.[17,18] Data were corrected for air and detector absorption, Lorentz and polarization effects[18] and scaled essentially by application of appropriate spherical harmonic functions.[18-20] Absorption by the crystal was treated numerically (Gaussian grid).[19,21] An illumination correction was performed as part of the numerical absorption correction.[19]

The structures were solved by ab initio dual space methods involving difference Fourier syntheses (VLD procedure, complex **2**),[22] by the heavy atom method (complex **3**)[22] or by the charge flip procedure (complex **4** · 2 *n*-hexane)[23] and refined by full-matrix least squares methods based on F^2 against all unique reflections.[24] All non-hydrogen atoms were given anisotropic displacement parameters. Hydrogen atoms were input at calculated positions and refined with a riding model. Split atom models were used to refine disordered groups and/or solvent molecules. When found necessary, suitable geometry and adp restraints were applied.[25] Due to severe disorder, electron density attributed to solvent of crystallization (*n*-hexane) was removed from the structures of **3** with the BYPASS procedure,[26] as implemented in PLATON (squeeze/hybrid).[27] Partial structure factors from the solvent masks were included in the refinement as separate contributions to F_{calc}.

CCDC 1967921 - 1967922 and 1985147 contains the supplementary crystallographic data for this paper. These data can be obtained free of charge from the Cambridge Crystallographic Data Centre's and FIZ Karlsruhe’s joint Access Service via https://www.ccdc.cam.ac.uk/structures/?.

S.7.2 STRUCTURE PARAMETERS

Table S9. Crystallographic data for **2, 3** and **4**.

	^{tBu}(PNP)Fe (**2**)	^{tBu}(PNP)Fe(OCPh$_2$) (**3**)	(^{tBu}(PNP)Fe)$_2$O · 2 *n*-hexane (**4** · 2 *n*-hexane)
formula	$C_{38}H_{62}FeNP_2$	$C_{51}H_{72}FeNOP_2$	$C_{88}H_{152}Fe_2N_2OP_4$
crystal system	monoclinic	monoclinic	monoclinic
space group	*I* 2/*a*	*P* 2$_1$/*c*	*C* 2/*c*
a /Å	23.5664(5)	13.04722(6)	23.0414(5)
b /Å	13.0211(2)	22.96022(12)	15.4342(3)
c /Å	25.5187(5)	15.94099(7)	25.5805(7)
β /°	106.794(2)	97.5543(4)	100.257(2)
V /Å^3	7496.7(3)	4733.95(4)	8951.7(3)
Z	8	4	4
M_r	650.67	832.88	1489.69
F_{000}	2824	1796	3256
d_c /Mg·m^{-3}	1.153	1.169	1.105
μ /mm^{-1}	4.203	3.453	3.583
max., min. transmission factors	0.836, 0.682	0.971, 0.664	1.000, 0.737
X-radiation, λ /Å	Cu-K_α, 1.54184	Cu-K_α, 1.54184	Cu-K_α, 1.54184
data collect. temperat. /K	120(1)	120(1)	120(1)
θ range /°	3.6 to 70.4	3.4 to 70.9	3.5 to 67.1
index ranges h, k, l	±28, ±15, ±30	±15, –27 ... 28, ±19	–27 ... 25, ±18, ±30
reflections measured	92210	249762	157390
unique [R_{int}]	7120 [0.0693]	9081 [0.0520]	8011 [0.2074]
observed [$I \geq 2\sigma(I)$]	6450	8320	3675
data / restraints /parameters	7120 / 0 / 397	9081 / 0 / 523	8011 / 48 / 402
GooF on F^2	1.034	1.038	0.944
R indices [$F>4\sigma(F)$] $R(F)$, $wR(F^2)$	0.0391, 0.1077	0.0282, 0.0686	0.0639, 0.1549
R indices (all data) $R(F)$, $wR(F^2)$	0.0441, 0.1117	0.0328, 0.0709	0.1498, 0.1953
largest residual peaks /e·Å^{-3}	0.450, –0.717	0.434, –0.311	0.357, –0.234
CCDC deposition number	1967921	1985147	1967922

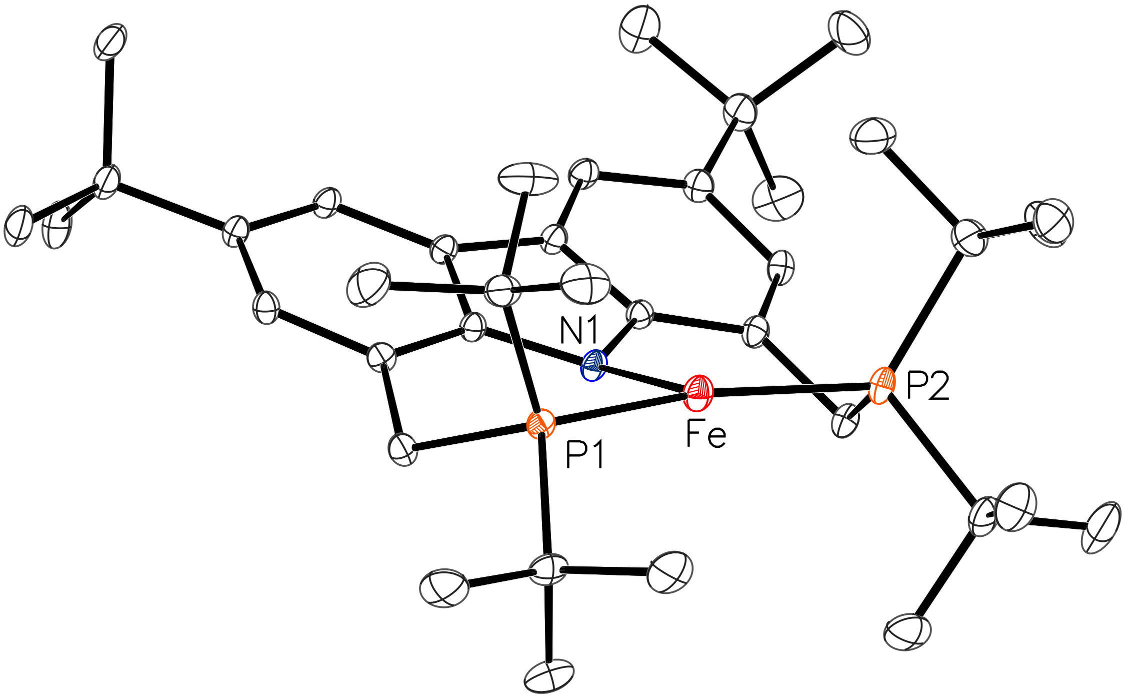

Figure S21. Molecular structure of ^{tBu}(PNP)Fe (**2**) with displacement ellipsoids drawn at 30 % probability. Hydrogen atoms are omitted for clarity. Selected bond lengths [Å] and angles [°]: Fe–P1 2.2680(6), Fe–P2 2.2853(6), Fe–N1 2.0369(16), N1–Fe–P1 96.22(5), N1–Fe–P2 96.26(5), P1–Fe–P2 166.68(2).

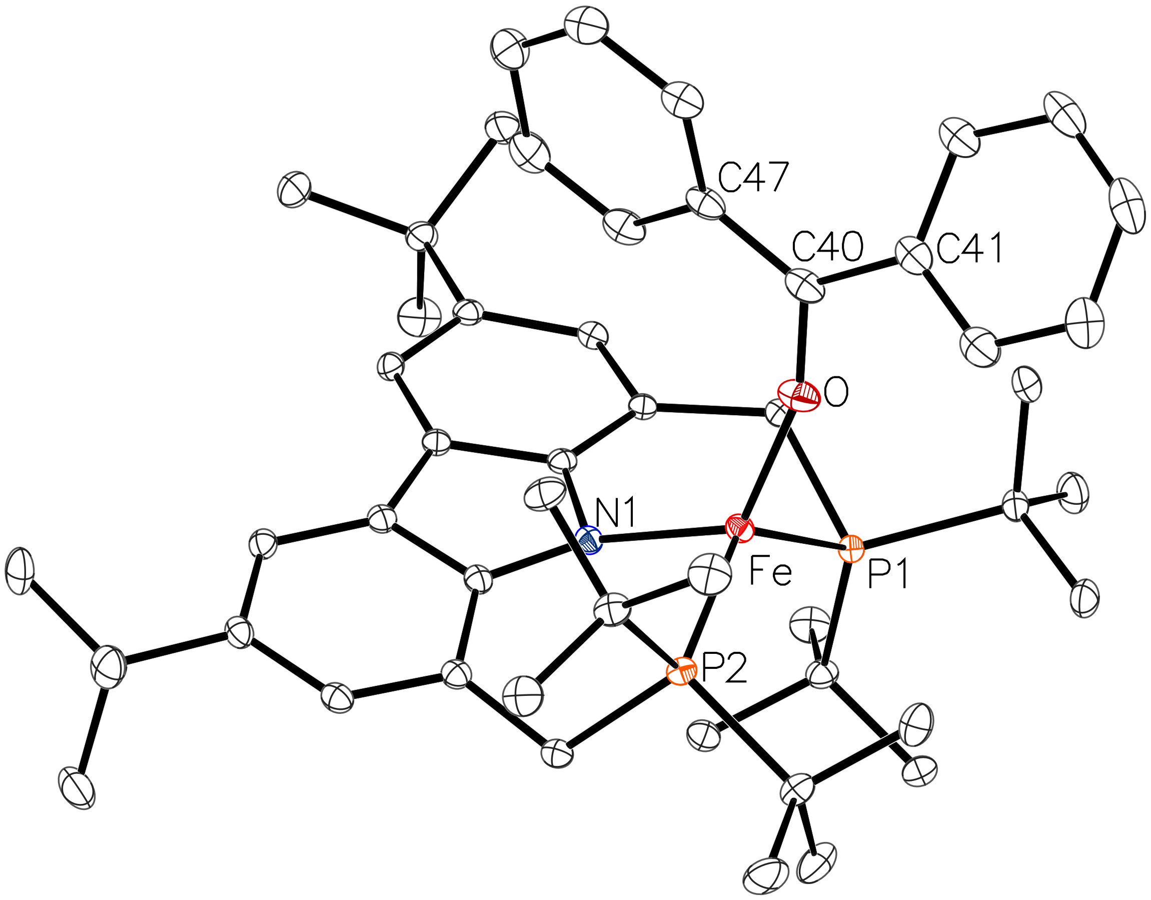

Figure S22. Molecular structure of ^{tBu}(PNP)Fe(OCPh$_2$) (**3**) with displacement ellipsoids drawn at 30 % probability. Hydrogen atoms are omitted for clarity. Selected bond lengths [Å] and angles [°]: Fe–P1 2.4599(4), Fe–P2 2.3834(4), Fe–N1 1.9816(11), Fe–O 1.8565(10), O–C40 1.2989(18), C40–C41 1.458(2), C40–C47 1.461(2), P1–Fe–P2 132.138(14), N1–Fe–P1 88.21(3), N1–Fe–P2 95.26(3), N1–Fe–O 127.75(5), Fe–O–C40 158.78(11), C41–C40–C47 124.84(14), O–C40–C41 115.63(14), O–C40–C47 119.53(13).

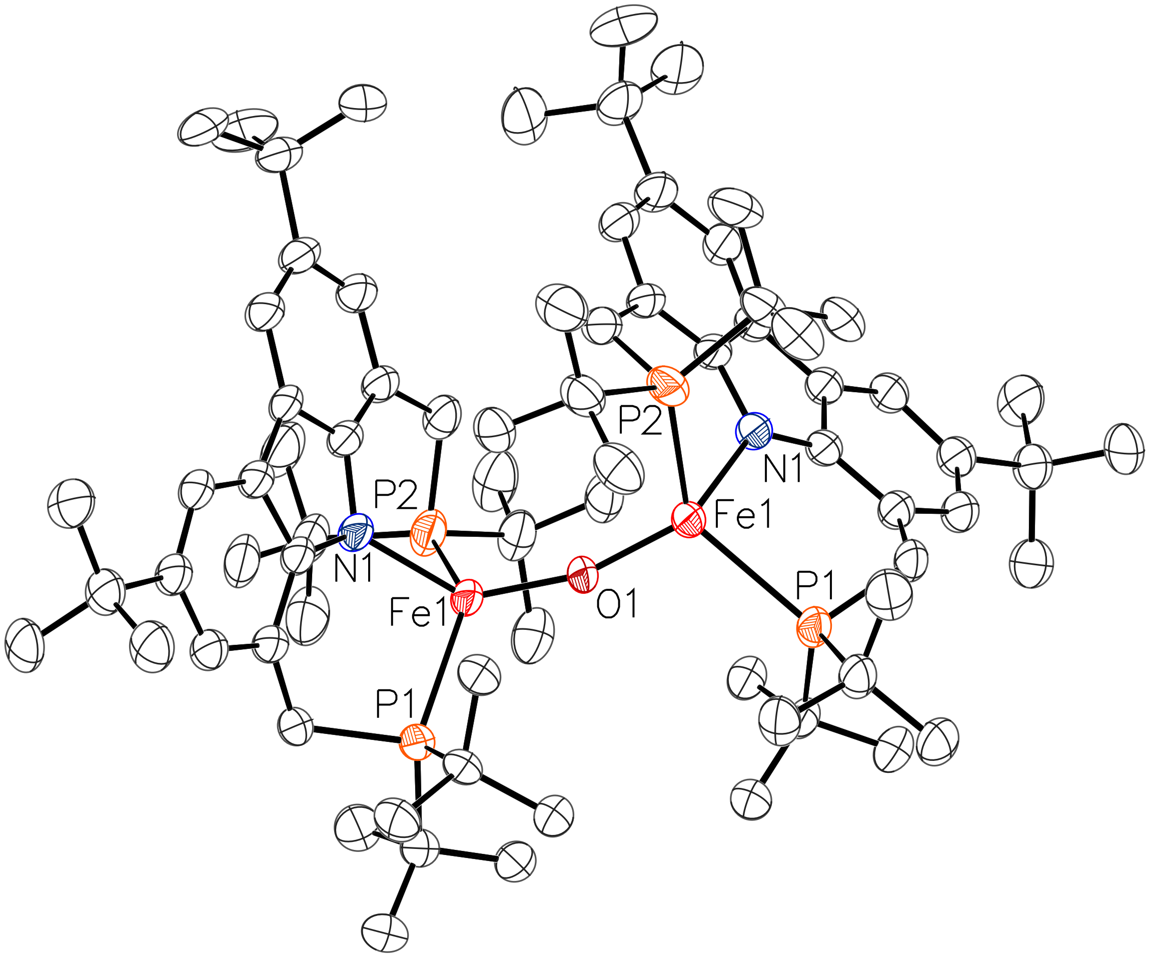

Figure S23. Molecular structure of (^{tBu}(PNP)Fe)$_2$O (**4**) with displacement ellipsoids drawn at 30 % probability. Hydrogen atoms are omitted for clarity. Selected bond lengths [Å] and angles [°]: Fe1–P1 2.4422(14), Fe1–P2 2.5323(16), Fe1–N1 2.012(3), Fe1–O1 1.7931(9), P1–Fe1–P2 127.24(6), O1–Fe1–P1 111.37(12), O1–Fe1–P2 110.81(8), O1–Fe1–N1 127.83(15), N1–Fe1–P1 92.91(11), N1–Fe1–P2 85.27(12), Fe1–O1–Fe1' 163.6(2).

S8 LITERATURE

[1] J. Higuchi, S. Kuriyama, A. Eizawa, K. Arashiba, K. Nakajima, Y. Nishibayashi, *Dalt. Trans.* **2018**, *47*, 1117–1121.
[2] I. Bertini, C. Luchinat, *Coord. Chem. Rev.* **1996**, *150*, 1–292.
[3] I. Bertini, C. Luchinat, G. Parigi, Eds. , *Solution NMR of Paramagnetic Molecules: Applications to Metallobiomolecules and Models*, Elsevier, New York, **2001**.
[4] I. Bertini, C. Luchinat, G. Parigi, E. Ravera, *Solution NMR of Paramagnetic Molecules*, Elsevier, Amsterdam, **2017**.
[5] M. Kaupp, F. H. Köhler, *Coord. Chem. Rev.* **2009**, *253*, 2376–2386.
[6] M. Kaupp, M. Bühl, V. G. Malkin, *Calculation of NMR and EPR Parameters*, Wiley-VCH, Weinheim, **2004**.
[7] J. A. Pople, *Mol. Phys.* **1958**, *1*, 175–180.
[8] H. F. Hameka, *Mol. Phys.* **1958**, *1*, 203–215.
[9] D. Zeroka, H. F. Hameka, *J. Chem. Phys.* **1966**, *45*, 300–311.
[10] K. Wolinski, J. F. Hinton, P. Pulay, *J. Am. Chem. Soc.* **1990**, *112*, 8251–8260.
[11] G. T. P. Charnock, I. Kuprov, *Phys. Chem. Chem. Phys.* **2014**, *16*, 20184–20189.
[12] H. J. Hogben, M. Krzystyniak, G. T. P. Charnock, P. J. Hore, I. Kuprov, *J. Magn. Reson.* **2011**, *208*, 179–194.
[13] M. J. Frisch, G. W. Trucks, H. B. Schlegel, G. E. Scuseria, M. A. Robb, J. R. Cheeseman, G. Scalmani, V. Barone, B. Mennucci, G. A. Petersson, et al., **2013**.
[14] B. Kirchner, F. Wennmohs, S. Ye, F. Neese, *Curr. Opin. Chem. Biol.* **2007**, *11*, 134–141.
[15] L. Noodleman, *J. Chem. Phys.* **1981**, *74*, 5737–5743.
[16] N. F. Chilton, R. P. Anderson, L. D. Turner, A. Soncini, K. S. Murray, *J. Comput. Chem.* **2013**, *34*, 1164–1175.
[17] K. Kabsch, in: M. G. Rossmann, E. Arnold (eds.) *"International Tables for Crystallography" Vol. F*, Ch. 11.3, Kluwer Academic Publishers, Dordrecht, The Netherlands, **2001**.
[18] *CrysAlisPro*, Agilent Technologies UK Ltd., Oxford, UK **2011-2014** and Rigaku Oxford Diffraction, Rigaku Polska Sp.z o.o., Wrocław, Poland **2015-2019**.
[19] *SCALE3 ABSPACK, CrysAlisPro*, Agilent Technologies UK Ltd., Oxford, UK **2011-2014** and Rigaku Oxford Diffraction, Rigaku Polska Sp.z o.o., Wrocław, Poland **2015-2019**.
[20] R. H. Blessing, *Acta Cryst.* **1995**, *A51*, 33.
[21] W. R. Busing, H. A. Levy, *Acta Cryst.* **1957**, *10*, 180.
[22] (a) M. C. Burla, R. Caliandro, B. Carrozzini, G. L. Cascarano, C. Cuocci, C. Giacovazzo, M. Mallamo, A. Mazzone, G. Polidori, *SIR2014*, CNR IC, Bari, Italy, **2014**; (b) M. C. Burla, R. Caliandro, B. Carrozzini, G. L. Cascarano, C. Cuocci, C. Giacovazzo, M. Mallamo, A. Mazzone, G. Polidori, *J. Appl. Cryst.* **2015**, *48*, 306.
[23] (a) L. Palatinus, *SUPERFLIP*, EPF Lausanne, Switzerland and Fyzikální ústav AV ČR, v. v. i., Prague, Czech Republic, **2007-2014**; (b) L. Palatinus, G. Chapuis, *J. Appl. Cryst.* **2007**, 40, 786.
[24] (a) G. M. Sheldrick, *SHELXL-20xx*, University of Göttingen and Bruker AXS GmbH, Karlsruhe, Germany **2012-2018**; (b) W. Robinson, G. M. Sheldrick in: N. W. Isaaks, M. R. Taylor (eds.) „*Crystallographic Computing 4*", Ch. 22, IUCr and Oxford University Press, Oxford, UK, **1988**; (c) G. M. Sheldrick, *Acta Cryst.* **2008**, *A64*, 112; (d) G. M. Sheldrick, *Acta Cryst.* **2015**, *C71*, 3.

[25] a) J. S. Rollett in: F. R. Ahmed, S. R. Hall, C. P. Huber (eds.) „*Crystallographic Computing*" p. 167, Munksgaard, Copenhagen, Denmark, **1970**; (b) D. Watkin in: N. W. Isaaks, M. R. Taylor (eds.) „*Crystallographic Computing 4*", Ch. 8, IUCr and Oxford University Press, Oxford, UK, **1988**; (c) P. Müller, R. Herbst-Irmer, A. L. Spek, T. R. Schneider, M. R. Sawaya in: P. Müller (ed.) *"Crystal Structure Refinement"*, Ch. 5, Oxford University Press, Oxford, UK, **2006**; (d) D. Watkin, *J. Appl. Cryst.* **2008**, *41*, 491.

[26] (a) P. v. d. Sluis, A. L. Spek, *Acta Cryst.* **1990**, *A46*, 194; (b) A. L. Spek, *Acta Cryst.* **2015**, *C71*, 9.

[27] (a) A. L. Spek, *PLATON*, Utrecht University, The Netherlands; (b) A. L. Spek, *J. Appl. Cryst*. **2003**, *36*, 7.
